# Sequence-Structure Analysis Unlocking the Potential Functional Application of the Local 3D Motifs of Plant-Derived Diterpene Synthases

**DOI:** 10.3390/biom14010120

**Published:** 2024-01-17

**Authors:** Yalan Zhao, Yupeng Liang, Gan Luo, Yi Li, Xiulin Han, Mengliang Wen

**Affiliations:** 1 National Key Laboratory for Conservation and Utilization of Bio-Resources in Yunnan, Yunnan University, Kunming 650091, China; ylzhao@mail.ynu.edu.cn (Y.Z.); lyp2019@mail.ynu.edu.cn (Y.L.); 12021214235@mail.ynu.edu.cn (G.L.); xlhan@ynu.edu.cn (X.H.); 2 Key Laboratory of Microbial Diversity in Southwest China, Ministry of Education, Yunnan Institute of Microbiology, School of Life Sciences, Yunnan University, Kunming 650091, China; 3 College of Mathematics and Computer Science, Dali University, Dali 671003, China

**Keywords:** plant diterpene synthases, functional annotation dataset, local structure motifs

## Abstract

Plant-derived diterpene synthases (PdiTPSs) play a critical role in the formation of structurally and functionally diverse diterpenoids. However, the specificity or functional-related features of PdiTPSs are not well understood. For a more profound insight, we collected, constructed, and curated 199 functionally characterized PdiTPSs and their corresponding 3D structures. The complex correlations among their sequences, domains, structures, and corresponding products were comprehensively analyzed. Ultimately, our focus narrowed to the geometric arrangement of local structures. We found that local structural alignment can rapidly localize product-specific residues that have been validated by mutagenesis experiments. Based on the 3D motifs derived from the residues around the substrate, we successfully searched diterpene synthases (diTPSs) from the predicted terpene synthases and newly characterized PdiTPSs, suggesting that the identified 3D motifs can serve as distinctive signatures in diTPSs (I and II class). Local structural analysis revealed the PdiTPSs with more conserved amino acid residues show features unique to class I and class II, whereas those with fewer conserved amino acid residues typically exhibit product diversity and specificity. These results provide an attractive method for discovering novel or functionally equivalent enzymes and probing the product specificity in cases where enzyme characterization is limited.

## 1. Introduction

Diterpenoid natural products belong to a class of widely distributed C20 isoprenoids, with more than 18,000 members identified in plants [1]. They play an important role in plant growth and development [2], mediate complex plant-environment interactions [3], and also have applications in medicine, flavor, and food industries [4,5,6,7]. All the discovered diterpenoids can be classified based on their core diterpene skeletons by removing all heteroatoms and stereocenters and reducing unsaturated structures [1,8].

Diterpenoids are highly diversified and complex compounds derived from the 5-carbon building blocks isopentenyl pyrophosphate (IPP) and dimethylallyl pyrophosphate (DMAPP). Geranylgeranyl pyrophosphate (GGPP) synthase catalyzes the coupling of IPP and DMAPP in a processive head-to-tail fashion to generate linear hydrocarbon molecules. Then, diTPSs and cytochrome P450 monooxygenases (P450s) are responsible for synthesizing a variety of intermediates and modifying skeletons [2,9,10,11]. In particular, diTPSs catalyze remarkably complex cyclization cascades with structural and stereochemical precision, creating a chemical library of 20-carbon hydrocarbons.

Based on the reaction mechanism, diTPSs employ either ionization-induced carbocation formation (diTPSs I), protonation-induced carbocation formation (diTPSs II), or use both mechanisms through bifunctional enzymes (diTPSs I/II) [12]. diTPSs II exist in the form of architectures with β, βγ, or αβγ domains, and their functional active site motif, DXDD, is located at the interface of the βγ domains where the substrate GGPP undergoes the reaction to generate intermediate [13,14]. diTPSs I exist in the form of architectures with α, αβ, or αβγ domains, housing the functional active site motif DDXXD in the α domain. In this region, the catalysis of products by diTPSs II results in diterpene precursors [13,14]. diTPSs I/II exist as fusion proteins with αβγ domains. Truncation studies failed to generate independently functional constructs of α (Class I) and βγ (Class II) domains, indicating that protein stability and structural integrity of each catalytic domain are largely dependent on crucial interactions at the α-β domain interface [15,16]. Detected during steady-state kinetic measurements, diTPSs II products diffuse in the solution, and then rebind to the active site of diTPSs I for the final cyclization reaction. This result contradicts the assumption of substrate channels between active sites [17]. Furthermore, the structural homology between the β and γ domains of squalene hopene cyclase and 23% amino acid sequence identity suggests the occurrence of ancestral gene duplication and fusion events, with catalytic activity evolving at the domain domain interface [18,19]. The available PdiTPS crystal structures indicate that most PdiTPSs contain up to three domains, namely γ, β, and α [15,20,21,22]. PdiTPSs I commonly feature non-active βγ domains and PdiTPSs II generally have an inactive α domain.

These diTPSs make a significant contribution to synthesizing a diverse range of diterpene skeletons. In plants, there are over a hundred known diterpene skeletons [1], yet the currently identified plant diterpene synthases can only synthesize less than one-tenth of the known skeletons. The limited knowledge of enzyme substrate recognition and product distribution of diTPSs hinders the identification of novel functional diTPSs. Classic multiple-sequence alignment methods have been used to identify the functional motifs of diTPSs. The identified functional sequence motifs include DXDD [23], DDXXD [24,25,26], NSE\DTE [27], PIX [28], and LHS...PNV [29,30,31]. Structural alignments also provide access to 3D motifs, which are typically formed by specific subsets of amino acid residues. The connections between these residues might not be readily apparent in the amino acid sequence, but they are consistently present in the three-dimensional spatial structure of the enzyme. These characteristics may be shared by the majority of enzymes, while at the same time, they can be unique to a specific enzyme class and relevant to its function [32,33]. They are characterized by the spatial proximity of multiple non-contiguous amino acid residues through the folding of the protein chain, resulting in the formation of a conserved or convergent spatial arrangement of structural modules.

Obtaining a substantial number of reliable structures to capture these 3D structural motifs was a challenging endeavor in the past. AlphaFold2 has generated reliable protein structures, helping to overcome the shortage of structural information. Its success in accurately predicting the three-dimensional (3D) folding of proteins and enzymes marks a revolution in structural biology. AlphaFold 2, along with its predicted structures, has found diverse applications, including studies of protein pockets [34], complex structure prediction [35,36], studies of structural similarity [37], and novel fold predictions [38]. It has predicted structures for billions of proteins, encompassing virtually all known proteins [39]. This has further spurred exploration into new protein families [40], discovering enzymes with new functions [41], and investigating the evolution and functionality of ancient proteins [42]. Structure-based method and the identification of functionally relevant motifs, we can delve into the multifunctional enzymes and the plasticity of functional sites. Ultimately, these studies will assist in delineating the 3D fingerprints of enzymes, providing crucial references for the exploration of uncharted protein functionalities. Currently, there are now techniques available to perform structural site comparisons, such as ProBis [43], PocketAlign [44], and SiteMotif [45]. These tools can align binding sites of interest in enzymes, paving the way for localized structural investigations into the functional diTPSs.

Furthermore, by examining the specific residues function relationship of *Selaginella moellendorffii* miltiradiene synthase (SmMDS), specific residues around the substrate responsible for product specificity, such as E690, S717, and H721, were identified [22]. The analysis of structures and catalytic mechanisms also suggests that the cavity formed by the substrate surrounding residues can selectively choose the substrate [46]. Product-changing mutational studies and structural analyses provide valuable insights for investigating PdiTPSs’ function. However, this research has only covered a small fraction of the characterized PdiTPSs. Furthermore, as of now, there has not been a comprehensive investigation of the PdiTPSs based on the sequence and structure.

Here, a manually curated and annotated database has been utilized to investigate the partitioning of PdiTPSs functions, using sequence similarity network (SSN) and phylogenetic analysis. The correlations were examined among various factors, including overall sequences, subsequences, overall structures, residues around the substrate, and product similarity. We calculated the residue preferences surrounding the substrate and analyzed their spatial conservation to identify the range of residues that significantly affect substrate type and product outcome. We analyzed the structural motifs around the substrates of PdiTPSs and used these motifs to search uncharacterized terpene synthases as well as recently characterized PdiTPSs. The comprehensive analysis here yielded valuable insights into exploring product-specific residues in PdiTPSs and the mapping patterns between PdiTPSs and their functions.

## 2. Materials and Methods

### 2.1. Collect Characterized PdiTPSs

To find potentially characterized PdiTPSs, we manually searched for experimental characterization of diterpenes from the literature up to May 2022 and collected their corresponding GenBank accession numbers (NCBI). Hidden Markov model (HMM) of the N-terminal domain (PF01397) and C-terminal domain (PF03936) of terpene synthases were downloaded from the Pfam database. Hmmsearch v.3.1.2 was used to search for the N- and C-terminal sequences in each PdiTPS sequence. When multiple N- or C-terminal sequences were identified, the result with the lowest *E*-value was retained (Tables S5 and S6).

### 2.2. Creation of Sequence Similarity Networks

All-versus-all pairwise local sequence alignments were performed using SSNpipe v.1.0.0 for PdiTPSs C, N, NC domains, and overall sequences [47]. The BLAST result files were searched with *E*-value thresholds ranging from 10^−5^ to 10^−140^ at 5 log unit intervals. The networks were visualized using Cytoscape (version 3.8.2).

### 2.3. Phylogenetic Analysis

The protein sequences were aligned using MAFFT v.7.310 [48], three methods of globalpair, gendfpair and localpair were used, and the bootstrap test was carried out with 1000 replicates. Manual inspection was performed to ensure proper alignment of known motifs such as the DDXXD and DXDD motif. Phylogenetic tree analysis was inferred using IQTree v.2.0.3 [49] with the following parameters: -s Mafft_Sequence -m JTT+F+R7 (full-length)/JTT+F+R5 (C-terminal and N-terminal subsequences) -B 1000 -nt AUTO. Levopimaradiene synthase from the hornwort *Phaeoceros carolinianus* was specified as the outgroup, and the tree was visualized by Chiplot [50].

### 2.4. Retrieve and Visualize Sequence Motifs

A total of 199 PdiTPSs amino acid sequences were submitted to the MEME online tool https://meme-suite.org/meme/tools/meme (accessed on 23 November 2022) to identify sequence motifs. Based on the width of functional motifs, the number of motifs was set to 20, and the minimum width of the motifs was set to 3. Other parameters were set to default values.

### 2.5. Calculations on Similarity and Correlation between Sequences, Structures, and Small Molecules

The similarities between C-terminal, N-terminal, and overall sequences were compared by using the TBtools [51] Protein Pairwise Similarity Matrix. The TM-align [52] was used to compare the topology similarities between the residues around the substrates and the overall structures, with the command “TMalign Pdb-A Pdb-B -outfmt 2″, and the TM-score value scales the structural similarity. The Dice Similarity Coefficient (DSC) [53] was used to measure chemical similarity in extended connectivity fingerprint (ECFP/Morgan Fingerprint, radius 2). The SMILES strings of the small molecules used in the calculations are provided in Table S4. For each pair of PdiTPSs, their corresponding products were arranged and combined, and the similarity score was calculated using the RDkit similarity matrix. For PdiTPSs that produce only one product, there is only one product pair and thus only one similarity value. However, for those that produce multiple products, several product pairs were obtained, and the average of their similarity scores was used to represent the product similarity values. The Pearson correlation coefficient (PPC) was calculated using the ggstatsplot package in R software (version 4.3.1) [54]. The correlation analysis involved the following factors: the overall sequences of 199 PdiTPSs, the sequence similarities of C-terminal, N-terminal, and NC terminal subsequences, as well as the structures formed by residues surrounding the substrates and the overall structures.

### 2.6. Structure Prediction and Molecular Docking

A total of 153 structures of PdiTPSs were downloaded from the AlphaFold database https://alphafold.com/ (accessed on 30 July 2022), and the rest were predicted for their 3D structures using ColabFold [55,56]. The obtained structures were docked with substrates using the CB-Dock2 molecular docking program https://cadd.labshare.cn/cb-dock2 (accessed on 11 September 2022) The parameters for molecular docking are the default parameters. The docking results generated by CB-Dock2 were selected to reference the crystal structures. Amino acids within a 4–10 Å range of the substrate were selected using the command “select AA, byres all within 4 of sele” in PyMOL (version 2.0) for further analysis. The diagrams were made with PyMOL.

### 2.7. Analyzing Amino Acid Frequencies and Preferences in the Proximity of Substrates

Software iLearnPlus v.1.0.1 [57] was used to extract the amino acid composition (AAC) and grouped amino acid composition (GAAC) features from the residues surrounding the substrates and the overall sequences. The resulting features included the frequencies of the 20 amino acids and their categorization based on 5 physicochemical properties (aliphatic, aromatic, negative charge, positive charge, uncharged). The residue preferential value was then calculated as the ratio of the frequency of each residue around the substrate to its frequency in the overall sequence.

### 2.8. Capturing Structure Motifs and Application

We utilized SiteMotif [45] to retrieve structural motifs generated by residues within 6 Å of the substrates for PdiTPSs that produce SK1-SK15, as well as motifs generated by residues within 8 Å of the substrates for PdiTPSs that produce different diterpene intermediates. The cutoff value with M-dist-min > 0.6 and M-dist-max > 0.4 to identify representative structural motifs in the SiteMotif. Subsequently, the retrieved motifs were visualized using PyMOL (version 2.0) and saved as aligned sequences. We further created Logo plots for these sequences using Hiplot (ORG) [58].

Additionally, we downloaded 342 putative terpene synthase structures from the AlphaFold2 protein structure database. Using the pyScoMotif (version 0.9.50) [59] to search for structural motifs in the uncharacterized terpene synthase structures and the newly identified structures of PdiTPSs. Utilizing the ‘—residue type policy = relaxed’ option to permit a maximum of one residue mutation while generating similar structural motifs.

## 3. Results and Discussion

### 3.1. Overview of Functional Annotations of PdiTPSs

We provide a curated database of 199 functional PdiTPSs, including 27 bifunctional enzymes, 90 class I enzymes, and 82 class II enzymes (Table S1). These PdiTPSs were derived from 69 plant species belonging to 26 families and 52 genera, producing 16 diterpene intermediates and 63 diterpene precursors. Of these products, only a small fraction was found to be associated with multiple PdiTPSs, while the majority of products were primarily catalyzed by a single PdiTPS, which affected the product-specific analysis.

To solve this problem, the existing terpenoid skeleton classification system [60] was employed to group these products into 16 different types. Products from PdiTPSs I and PdiTPSs I/II were classified into 15 skeleton types, while those from PdiTPSs II were classified into only a single type based on the preserved phosphate group features (Table S1). Detailed information about the structures of the products and substrates, as well as the grouping of backbone structures, are provided in supplementary material (Tables S2 and S3). The classification scheme allowed us to group multiple products from a single enzyme into the same category, such as SsSS synthase from *Salvia sclarea* [5], which could catalyze the dephosphorylation and minor rearrangement of 9 diterpene intermediates to produce 11 diterpene precursors, all of which fall into the same labdane scaffold. However, this classification system is not always effective; for example, TrTPS13 from *Tripterygium regelii* produces five products belonging to pimarane, kaurane, and stemodane scaffolds (ntkrn, sndarpardn, ipsfdn, spsfdn, sdmon), and PdiTPSs from *Grindelia hirsutula* produces three products (abedn, epmnlo, mnlo) belonging to labdane and abietane scaffolds, respectively (Table S3). Therefore, in this study, we also attempt to explore the correlation between PdiTPSs in terms of sequence and structure with substrates or products’ similarity. Although the diversity of products and high substrate promiscuity challenge the analysis of enzyme product specificity, these enzymes can be used to explore and obtain new diterpenes. For example, Peter and his colleagues established a new biosynthetic pathway of 16 diterpenes using extreme promiscuity diTPSs from plant SsSS (*Salvia sclarea*) and bacterial KgTS (*Kitasatospora griseola*) [61]. Subsequently, they used the heterozygosity of diTPSs for combinatorial biosynthesis in their research. Their work offers novel biosynthetic access to almost 19 labdane-related diterpenes, showcasing the power of the combinatorial approach for expanding chemical diversity and potential application [62].

### 3.2. The SSN Topology Displays Fuzzy Functional Relationships of PdiTPSs

Applying a skeleton classification system for functional mapping in SSN analysis was examined. This all-pairs local sequence-based comparison method could rapidly generate a network of nodes and edges using any expectation value (E) as a threshold. SSN can be used to assign functions to uncharacterized enzymes, such as exploring the functional relationship between the glutamylation domain of the lantibiotic dehydratases [63] and achieving functional partitioning of sesquiterpene synthases, correctly predicting new sesquiterpene synthases from five different fungi [64].

**Figure 1:**
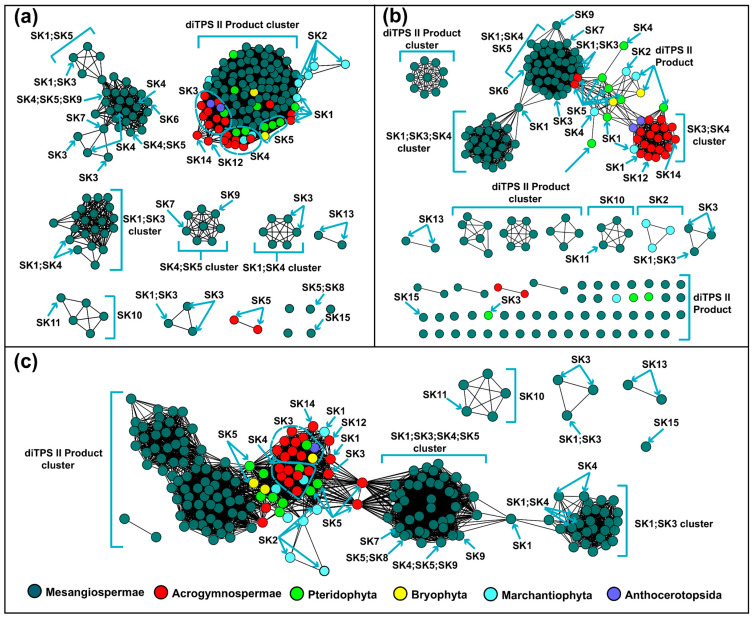
Similarity networks of PdiTPSs sequences. (**a**) Clusters of PdiTPSs product skeletons defined by N-terminal subsequence similarity (*E*-value threshold 10^−70^). (**b**) Clusters of PdiTPSs product skeletons defined by C-terminal subsequence similarity *(E*-value threshold 10^−70^). (**c**) Clusters of PdiTPSs product skeletons defined by NC-terminal subsequence similarity (*E*-value threshold 10^−120^).

The analysis above suggests that C-terminal, N-terminal, NC-terminal subsequences, and overall sequences can be used for classifying PdiTPSs I and PdiTPSs II. In general, the N-terminal (Figure 1a), C-terminal (Figure 1b), and NC-terminal (Figure 1c) networks generated by SSN mainly resulted in multiple backbone clustering. In particular, labdane, abietane, and pimarane skeletons were often clustered together, despite the clear differences in their product structures. In contrast, the product skeleton clusters obtained from multiple sequence comparisons of N-terminal subsequences were more refined, with fewer outliers.

While PdiTPSs from different species clustered together based on multiple sequence alignments, grouping them according to products has limitations. Moreover, we observed that the most abundant and widely distributed skeletons, such as labdane, abietane, pimarane, and kaurane [65] are predominantly produced by PdiTPSs from early diverging plant lineages, such as ferns and mosses (Figure 1). Further insights into the relationship between the product backbones and enzyme sequences were from SSN analysis. We found that the labdane and kaurane skeletons serve as crucial linkage points for other skeleton groups (Figure 1c). This implies that they may be the fundamental skeletons driving the evolution and diversification of diTPS products, but further experimental validation is required to confirm this hypothesis.

### 3.3. The Functional Subgroups of PdiTPSs Remain Unclear from a Phylogenetic Perspective

The phylogenetic tree of PdiTPSs was constructed to detect evolutionary relationships and identify lineages with similar features. By examining the change in product skeletons, it should be possible to identify the potential correlation between products and PdiTPSs mapping. Terpene synthases commonly contain two conserved structural domains, the N-terminal and C-terminal domains. These two structural domains comprise active sites, and in the analysis of product specificity in plant sesquiterpene synthases, isolated C-terminal subsequence characteristics have been found to effectively explain product specificity [66,67]. Therefore, phylogenetic trees for N-terminal and C-terminal subsequences were constructed.

**Figure 2:**
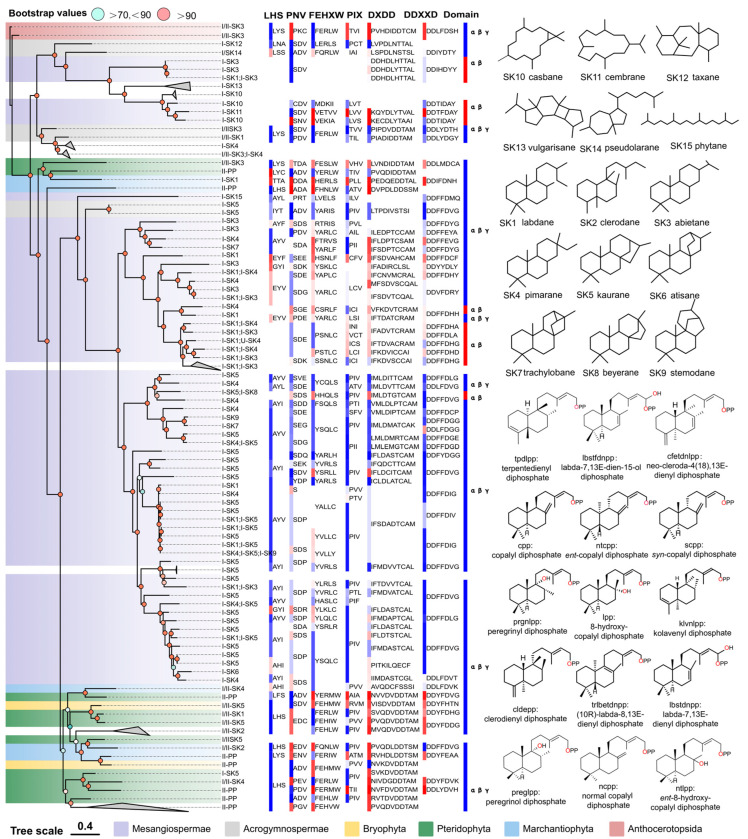
The relationship between the overall sequence phylogeny of PdiTPSs, their product scaffolds, function-related motifs, and biosource classification. The phylogenetic tree labels each enzyme with its accession ID, product class, and scaffold classification. It also displays the skeleton structures of SK1-SK15, product structures of PdiTPSs II, function-verified motifs in some PdiTPSs, and the domain composition of each PdiTPSs. The color blocks of motifs and domains in the figure only distinguish the continuous conserved motifs and domains. The color blocks in the PdiTPSs biosource classification represent the same phylum.

Unfortunately, the phylogenetic tree did not provide a clear division of PdiTPSs based on their functions. However, the labdane, clerodane, pimarane, kaurane, and PdiTPSs II products were frequently present in the early diverging PdiTPSs products. While this phylogenetic tree exclusively illustrates the gene’s evolutionary relationships, similar patterns are also evident in the phylogenetic trees of the overall sequences (Figure 2), as well as the N-terminal (Figure S1A) and C-terminal (Figure S1B) domains. The atisane, trachylobane, beyerane, and stemodane were found in the late-emerging PdiTPS products. There were observed trends in functions of PdiTPS products, towards evolving multi-ring skeletons from the major branches of the trees. Moreover, the PdiTPSs that produced the casbane, cembrane, taxane, vulgarisane, and pseudolarane skeletons showed shorter evolutionary distances from the ancestral PdiTPSs. This provides valuable insights into how the function and evolution of PdiTPSs may contribute to species-specific adaptations to unique ecological niches. However, additional investigations are necessary to further explore the distribution patterns of diterpenoid compound types and their relationship with the evolutionary status of plants. Similar research has been carried out to investigate the distribution of terpenoid compounds and their biosynthetic pathways in various species of *Isodon* plants [68,69].

The domain composition of PdiTPSs showed that the γβα triple-domain structure and βα bi-domain structure alternately appeared in the phylogenetic tree, indicating a phenomenon of continual loss and acquisition of structural domain subsequences during the evolution of terpene synthases. Additionally, the bi-domain βα structure, seen only in angiosperms (Figure 2), originated from the loss of the γ domain in ancestral terpene synthases with the γβα structure [13,69].

The LHS and PNV motifs (Figure 2) were copalyl diphosphate synthase (CPS)-specific motifs [29], as confirmed by sequence-based analysis. These two motifs were conserved in PdiTPSs II, but had undergone mutations in PdiTPSs I. The histidine (H) residue in the FEHXW motif exerted cooperative GGPP/Mg^2+^ inhibition on CPS [70], but histidine was not always conserved in the FEHXW motif of PdiTPSs II. Although the function of aromatic amino acids in this motif remained unclear, apparently in PdiTPSs I these residues were no longer predominantly composed of aromatic amino acids in this motif, but rather of aliphatic and uncharged amino acids. The PIX motif (Figure 2) displayed was related to *ent*-kaurene [28] and was lost in the PdiTPSs of angiosperms that produced primarily labdane and abietane, as well as in that of polycyclic skeleton casbane, cembrane, and vulgarisane. This motif was present in the PdiTPSs of mosses that produce labdane and abietane, but had undergone mutations. This suggests that potentially product-specific motifs might exist in PdiTPSs, which have evolved through deletions and mutations, and thereby resulted in enzyme sequences with newly acquired functions. Motifs therefore are different from those in other PdiTPSs or being absent in other PdiTPSs may help uncover product-specific motifs.

### 3.4. PdiTPSs with Conserved N-Terminal and Variable C-Terminal Subsequences

**Figure 3:**
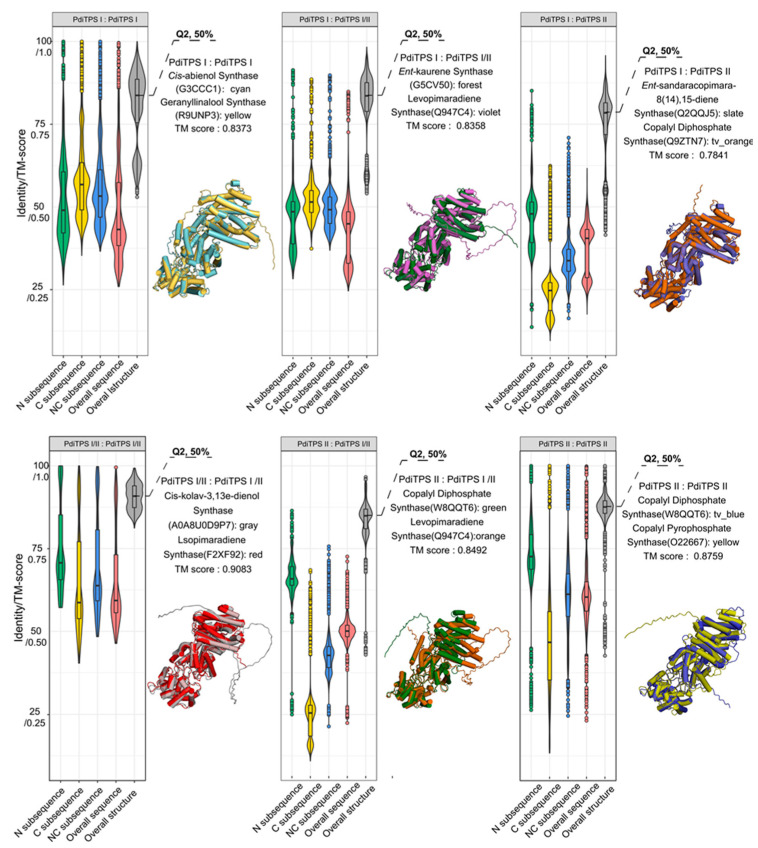
Distribution of sequences and structural similarities and representative structure (Q2, 50%). Distribution of sequences and structural similarities among N-terminal, C-terminal, NC-terminal sequences, overall sequences, and overall structures of PdiTPSs I, II, and I/II. Superimposition of representative structures at the Q2 position based on the TM-score. The same color in the distribution of sequence and structural similarities represent the same sequences and structure types, respectively.

The box plot was used to visualize the sequence similarity features of PdiTPSs. Larger values for the upper quartile and lower quartile in the box plot indicate higher similarity among sequences. Statistical results (Figure 3) showed that the C-terminal was more conserved in PdiTPSs I, while the N-terminal was more conserved in PdiTPSs II. The main differences in sequence similarity between PdiTPSs I and PdiTPSs II were located in the C-terminal (Figure 3), suggesting that the use of C-terminal subsequences might facilitate divergence. The similarity distribution of PdiTPSs I and PdiTPSs I/II sequences was lower in the NC-terminus and overall regions than that of in the C- and N-terminal subsequences, while the similarity distribution of PdiTPSs II and PdiTPSs I/II sequences was lower in the C-terminus than that of in the N-, NC-, and global sequences (Figure 3).

**Figure 4:**
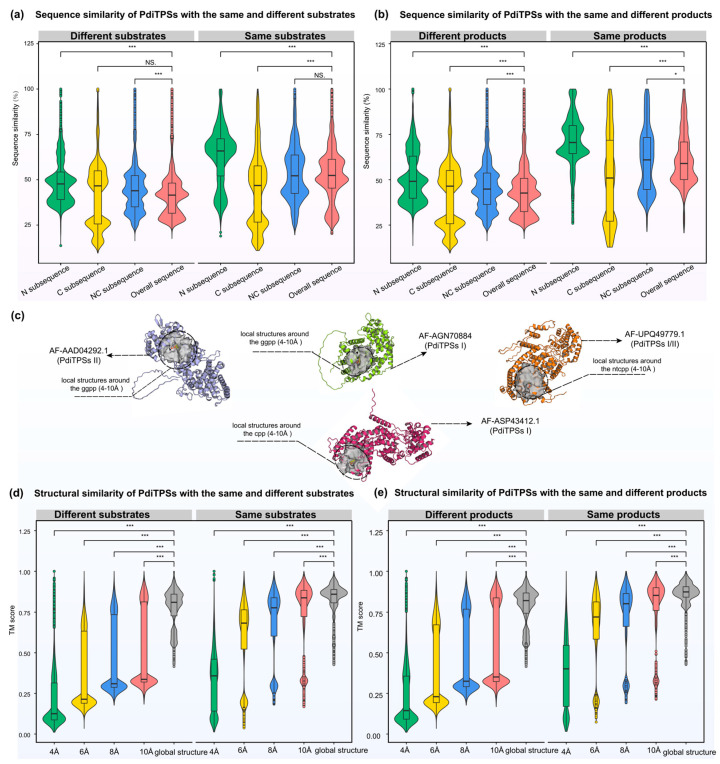
Distribution of similarities between N-terminal, C-terminal, NC-terminal subsequences, overall sequences, and overall structures of PdiTPSs for the same and different substrates and products. (**a**) Comparison of sequence similarities between N-terminal, C-terminal, and NC-terminal subsequences and overall sequences for the same and different substrates. (**b**) Comparison of sequence similarities between N-terminal, C-terminal, and NC-terminal subsequences and overall sequences for the same and different products. (**c**) The representative display of the range of positions of residues around the substrate under analysis. (**d**) Comparison of structure fold similarities between the local structures formed by residues within 4 Å, 6 Å, 8 Å, and 10 Å of the substrates with the overall structures for the same and different substrates. (**e**) Comparison of fold similarities between the local structures formed by residues within 4 Å, 6 Å, 8 Å, and 10 Å of the substrates with the overall structures for the same and different products. Asterisks indicate statistical significance (*: *p* < 0.05, ***: *p* < 0.001), and “NS” indicates no statistical difference. The same color in (**a**,**b**) represents the same sequence types. The same color in (**d**,**e**) represents the same local structure types.

After obtaining the basic conservation features of the PdiTPSs sequences, we compared and assessed sequence similarity in terms of substrate recognition and product generation. Theoretically, sequence similarities that are required to recognize the same substrates or to produce the same products, should be higher than that which recognizes different substrates or produces different products. The results are consistent. The N-terminal subsequences had the highest upper and lower quartiles of sequence similarities in identifying the same substrates and producing the same products. In contrast, the C-terminal subsequences had the lowest lower quartile of sequence similarities in identifying different substrates and producing different products. Additionally, the C-terminal subsequences presented the fewest 100% sequence similarity values in identifying different substrates and producing different products (Figure 4a,b). Similarly, sesquiterpene synthases have been reported to construct a phylogenetic tree using the C-terminal subsequences, which can group enzymes based on their product types [66]. This is also reflected in the SSN analysis, where the C-terminal subsequences generated more different clusters and distant outliers for different types of products than the N-terminal subsequences at the same threshold (Figure 1a,b).

### 3.5. PdiTPSs Have a Conserved Global Structure and a Flexible Fold of Surrounding Residues

While some clues have been obtained from sequences to predict the substrates and products of PdiTPSs, these have not yet expanded our insights into the functional features. Hence, further examination of PdiTPSs’ structures and their substrate-surrounding residues is warranted. The distribution of fold similarities for the global structures has been calculated (Figure 3), with most TM scores exceeding 0.75. Furthermore, the median TM scores of the overall structures when recognizing the same and different substrates (Figure 4d), as well as producing the same and different products (Figure 4e), were also greater than 0.75 and closer to the upper quartile (Q3). These results suggested that PdiTPSs that perform different functions shared a similar TPS fold. Here, only the superimposed results of the representative structures of different types of PdiTPSs at the median (Q2) of TM scores were shown (Figure 3). Supplementary Figure S2 showed the global structural superposition results of the representative structures of different types of structures representing PdiTPSs in Figure 3 with 2 extreme values and 3 quartiles of TM score. Furthermore, increasing the selected residue range around the substrate led to a higher TM score, signifying greater differences in the local structure formed by residues closer to the substrate. Figure 4c displays the location of the local structure constituted by the residues surrounding the substrate. Conversely, the conservation of the local structure formed by residues farther from the substrate increased, although the rate of increase leveled off (Figure 4d,e).

Both the local structures formed by substrate-surrounding residues and the overall structures exhibited significantly higher TM scores when compared to those of different substrates or different products (Figure 4d,e). This trend was more significant than the overall difference trend contributed by sequence similarity (Figure 4a,b). The results of structural analysis are similar to the evaluation of sequence similarity. The TM scores between the residues around the substrates and the whole structures that recognize the same substrates or produce the same product are greater than 0.5. Conversely, the TM scores for those structures recognizing different substrates and producing different products would be less than 0.5. Therefore, the local structures formed by residues within 6 Å of the substrates appeared to be the best choice for determining substrate and product similarity.

### 3.6. N-Terminal Subsequence Strongly Correlates with Overall Sequence Similarity

We have provided some insights for the determination of product types at both the sequence and structure levels. Hence, it would be interesting to further explore methods for quantitatively assessing the relationship between PdiTPSs sequences, structures, and products. The correlation was evaluated using Pearson’s correlation coefficient (PCC) [71], with only the final coefficient being shown here. The statistical results showed that the similarity between the C-terminal subsequences and the overall sequences (PCC = 0.46, *p* < 0.001) was significantly weaker than that between the N-terminal subsequences (PCC = 0.91, *p* < 0.001). And there was almost no correlation between the C-terminal and N-terminal subsequences (PCC = 0.26, *p* < 0.001).

The weak correlation of sequence similarities between C-terminal and N-terminal indicates that their contributions to PdiTPSs specificity are indeed significantly different. However, the phylogenetic trees of the C-terminal subsequence, N-terminal subsequence, and full-length sequence are similar. Another noteworthy observation is that the sequence similarity between the N-terminal subsequence and the full-length sequence is highly correlated, while the C-terminal subsequence is not related to either the N-terminal or the full-length sequence. A study has suggested that the N-terminal subsequence has been maintained conservation during evolution [14]. In contrast, the C-terminal subsequence likely underwent functional selection and evolved at a faster rate in order to acquire new functions to adapt to the environment. Clues to this can be found in our statistics of the sequence divergence of the C-terminal subsequences than the N-terminal subsequences.

### 3.7. Substrate-Surrounding Residue Topology in PdiTPSs Is Independent of the Overall Structure

The analysis based on sequence features provides limited insights into substrate recognition and functional diversification of PdiTPSs. Protein structures provide a higher resolution platform for understanding function, but acquiring protein structures is expensive and difficult. The emergence of AlphaFold2 and its high accuracy is exciting, as it has been applied to understand the mechanisms of enzymes [72]. AlphaFold2 has been applied to obtain structural data for the PdiTPSs in this study. Blind docking using CB-Dock2 can be used to reveal key residues that are functionally relevant in the binding pocket [73,74,75]. Based on the structural data, it has been observed that the variable arrangement of the γ, β, and α domains in PdiTPSs (Figure 2) is an important strategy for expanding and diversifying diTPSs. Their combination, presence, and absence constitute the structural chemistry of diTPSs [14,15,21].

In addition, the correlation analysis showed that the similarity of the overall structure increased with the similarity of the overall sequence (PCC = 0.78, *p* < 0.001). The N-terminal subsequence showed a high correlation with the overall sequence and a moderate correlation with the TM score of the overall structure (PCC = 0.69, *p* < 0.001). Conversely, the C-terminal subsequence differed significantly from the N-terminal and overall sequence and exhibited weak correlation with the overall structure (PCC = 0.33, *p* < 0.001). When analyzing the correlation between residues surrounding the substrate and the overall structure, the trend was completely opposite (Table S9). The average correlation between the C-terminal subsequence (0.54) and residues around the substrate was higher than that between the N-terminal subsequence (0.37). Combining the N- and C-terminal subsequence as the NC-terminal subsequence increased the average correlation with residues around the substrate to 0.58, which is understandable because both N- and C-terminal subsequence contain residues around the substrate. Furthermore, there was no correlation between residues around the substrate and the topology of the overall structure, as indicated by the TM score distribution. This distribution was significantly lower for residues around the substrate compared to the overall structure (Figure 4d,e). This indicates that the local structures of PdiTPSs, formed by residues around the substrates, may have significantly different folding mechanisms compared to the overall structures.

To evaluate the relationship between sequence similarity, the TM scores of protein structures, and products, the impact of sequence similarities, and the TM scores of products were indirectly calculated by measuring the strength of their correlations. The final correlation coefficients between product similarities and sequences and structures are summarized in Table 1. Surprisingly, the overall sequence had the highest correlation with the product, while the residues around the substrate and the overall topology had a weak correlation with the product. The phenomenon can be explained, as our research primarily centers around assessing the geometric compatibility between the substrate and the enzyme pocket. It is evident that, in the catalytic process of diTPSs, the geometric selection of substrate by the enzyme is only one of the factors. Hence, we propose a deeper exploration of the conserved physicochemical properties of residues occupying the same spatial vicinity to the substrate. This avenue of investigation promises a more insightful elucidation of the intricate interplay between diTPSs and the process of product formation.

**Figure 5:**
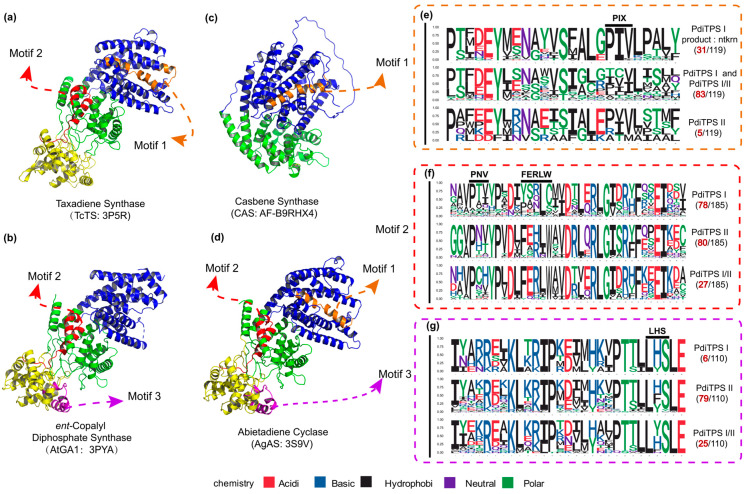
The positions of validated structure, along with the number of motifs and specific residues near the motifs in the three classes of PdiTPSs. (**a**) The crystal structure of Taxadiene synthase (diTPSs I) [20]. (**b**) The crystal structure of *ent*-copalyl diphosphate synthase (diTPSs II) [21]. (**c**) The crystal structure of Casbene synthase (diTPSs I). (**d**) The crystal structure of Abietadiene cyclase (diTPSs I/II) [15]. (**e**) Seqlogo of the PIX motif and its neighboring residues. (**f**) Seqlogo of the PNV and FERLW motifs and their neighboring residues. (**g**) Seqlogo of the LHS motif and its neighboring residues. The black numbers represent the total number of sequences retrieved by MEME for the PNV, FERLW, LHS, and PIX motifs. The red numbers represent the number of occurrences of each motif in the three classes of PdiTPSs. In (**a**–**d**), the colors yellow, green, and blue, respectively, represent the γ, β, and α structural domains.

To further validate the impact of the correlation between product and sequence similarity on product grouping, the similarity mapping between product and conserved motifs in PdiTPSs was analyzed by MEME. The four signature motifs (LHS, PNV, FERLW, and PIX) located in one differently long motif (Figure 5) have been identified. The similarities of these motifs to the products were then correlated with the similarities of the products. The correlation between the similarities of motifs 2 (PNV and FERLW) and products was 0.51, while the correlations for motif 1 (PIX) and motif 3 (LHS) were 0.32 and 0.36, respectively. Motifs showing stronger product correlations exhibit fewer mutations in unfunctional motifs and vice versa. This correlation indirectly reflects the differentiation level among enzyme motifs. Motifs with high product correlation likely represent functional domains of the PdiTPSs, whereas positions with low product correlation may contain product-specific motifs. Additionally, these functionally identified motifs, except for DDXXD and DXDD, can be aligned across all diTPSs and easily identified. However, the remaining motifs can only be discovered in enzymes with the same functions. After aligning enzymes with different functions, these motifs are overshadowed (Figure 5e–g). Yet, evidently, these motifs have fixed positions in the structures (Figure 5a–d). This phenomenon further emphasizes our focus on structural features.

### 3.8. Aromatic Residues around PdiTPSs Substrates Affect the Substrate Selection

**Figure 6:**
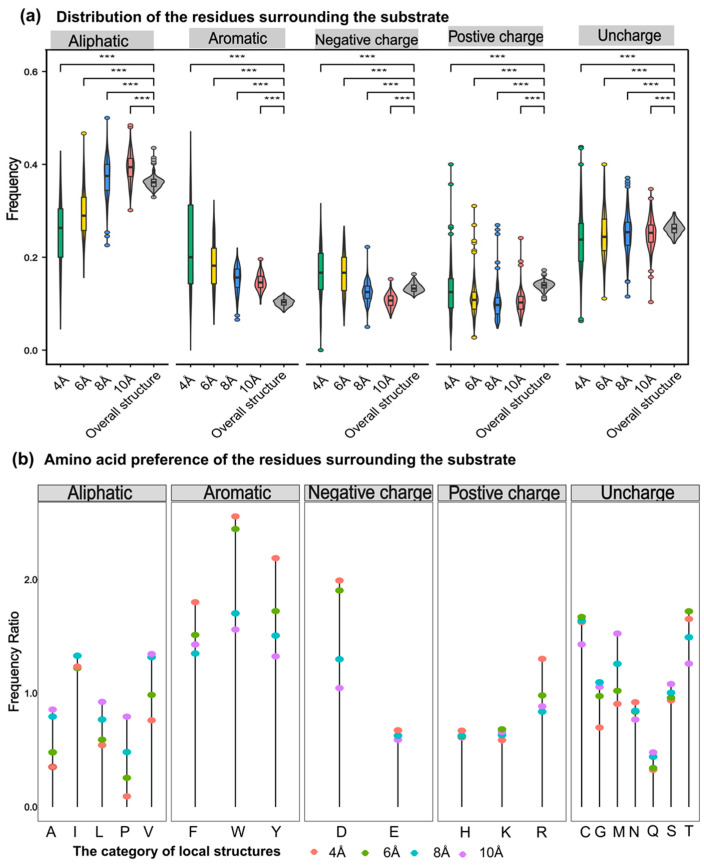
Preference of 5 types of amino acids in the residues around the substrate. (**a**) Frequency distribution of five types of amino acids (aliphatic, aromatic, negative charge, positive charge, and uncharged) within 4 Å, 6 Å, 8 Å, and 10 Å from the substrate compared to their overall frequency within the structure. (**b**) Comparison of the frequency of 20 amino acids within the structure to their frequency within 4 Å, 6 Å, 8 Å, and 10 Å from the substrate. Asterisks indicate statistical significance (***: *p* < 0.001), and “NS” indicates no statistical difference.

**Figure 7:**
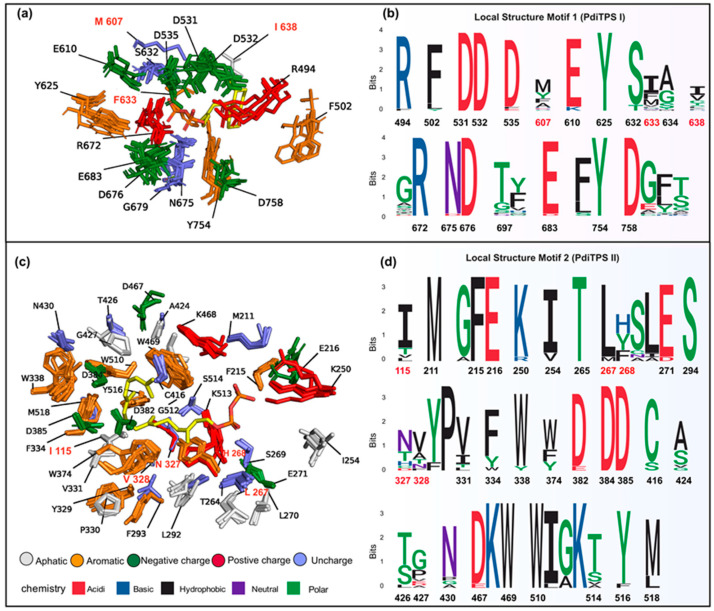
(**a**) Structurally aligned representative residues (within 6 Å) around the substrates of PdiTPSs (I and I/II) producing different backbones are shown superimposed. Residues labeled *ent*-kaurene synthase (AtKSL:AF-AAC39443). (**b**) The residue conservation corresponds to the positions depicted in (**a**). (**c**) Structurally aligned representative residues (within 8 Å) around the substrates of PdiTPSs (II and I/II) producing different intermediates are shown superimposed. Residues labeled *ent*-copalyl diphosphate synthase (TwTPS3:AF-ANO43020). (**d**) The residue conservation corresponds to the positions depicted in (**c**). In (**b**,**d**), aromatic residues and residues that have been shown to affect product outcome in mutagenesis studies are highlighted as red fonts indicate mutated residues.

Generally, the probability of catalytic-associated residues appearing elsewhere in the sequence should be lower than that of appearing at the catalytic center. The aromatic amino acids around substrates with different sizes were the most frequently observed in PdiTPSs (Figure 6a,b). This may be due to aromatic rings in aromatic amino acids providing electrons to stabilize carbocation intermediates in the PdiTPSs catalysis, facilitating substrate conversion. Specifically, tryptophan (W) was more likely to occur around the linear substrate of GGPP, while phenylalanine (F) and tyrosine (Y) were more likely to present around the substrate of the initial cyclization intermediates of PdiTPSs (Figure 7b,d).

The aromatic residues of PdiTPSs at the active site usually contain at least 2–3 aromatic residues, which most likely guide the intermediate involved in the reaction through spatial constraints and cation-π interactions. Moreover, the number of aromatic residues in the active site can be used to predict the promiscuity of the enzyme [76]. However, this report explains the selectivity of the observed substrate structures on the observed types of aromatic residues.

### 3.9. The Spatial Landscape of Conserved Residues and Less Conserved Residues around PdiTPSs Substrates and Their Applications

The substrate-surrounding residues of PdiTPSs include those that have been experimentally verified impacts on product formation. Note that the physicochemical properties and spatial orientations of these residues surrounding the substrate were highly conserved. Some frequently occurring residues, such as arginine (R), cysteine (C), tryptophan (W), aspartic acid (D), isoleucine (I), serine (S), threonine (T), valine (V), phenylalanine (F), tyrosine (Y), and methionine (M) (Figure 6b), were also conserved in their spatial positions (Figure 7c,d). Except for arginine (R), cysteine (C), phenylalanine (F), and tryptophan (W), the effects of the other residues on enzyme function and product had been demonstrated by mutagenesis experiments. For example, the PdiTPSs OsKSL5i: I664T and OsKSL5i: I718V from rice (*Oryza sativa*) specifically produced *ent*-pimara-8(14),15-diene and *ent*-isokaur-15-ene, respectively [77]. Six PdiTPSs from *Tripterygium wilfordii* independently evolved new functions by mutating specific residues, including TwKSL1v2: M607\T638A, TwKSL3: M608\I639, TwKSL2: A608\I639, TwCPS3: I115\N327\V328\H268, TwCPS5: T115\A327\T326, and TwCPS6: Y265 [78]. Moreover, mutation of glutamic acid (E) at position 690 to arginine (R), phenylalanine (F), lysine (K), proline (P), or aspartic acid (D) or mutation of serine (S) at position 721 to valine (V) in SmMDS resulted in product loss [22], respectively.

Low-frequency residues, including alanine (A) and histidine (H), also contribute to product specificity. For example, the AgAS: A723S mutant of abietadiene synthase specifically produced pimaradienes, and the H268 residue in TwCPS3 also contributed to product specificity [78,79]. Additionally, these low-frequency residues around the substrate often appeared spatially conserved but less conserved in physicochemical properties (Figure 7c,d). Mutated residues affecting the products mainly occurred within 6 Å of the substrates in PdiTPSs I (Figure 7a) and within 8 Å of that in PdiTPSs II (Figure 7c). For specific residue shapes and maps within 4 Å to 8 Å of representative PdiTPSs I and PdiTPSs I/II that produced SK1-SK15 skeleton types, as well as that of PdiTPSs II, please refer to the Supplementary Materials (Figures S3–S23).

The impact of residues surrounding the substrate on product outcomes has also been observed in other enzymes. For example, the residues around the substrate in P450 enzymes can control regio- and stereo-specificity in the biosynthesis of bacterial heterodimeric diketopiperazines [80]. The local structural analysis reveals that exploring and examining local structural alignment to generate sequence-order independent structural site motifs [45,81] might offer us a perspective to unveil the remarkable chemical diversity of PdiTPSs.

Given our observation of conservative spatial arrangements among certain residues around the substrates of PdiTPSs, we employed SiteMotif to separately search for the existence of conservative structural motifs within 8 Å and 6 Å of residues that generate different diterpene scaffolds and intermediates. This method allows for the rapid batch retrieval of structural motifs within multiple local structures and has been proven effective in obtaining motifs of binding sites for glutathione-binding proteins [45]. Based on the results retrieved by SiteMotif, we observed significant differences in local structural motifs of PdiTPSs for producing various diterpene scaffolds (Figure 7b) and intermediates (Figure 7d) in PdiTPSs. Considering the inclusion of diverse characteristics in PdiTPSs, we hypothesize that these two motifs may represent the characteristic site atlas of residues around the substrates of PdiTPSs. Then, pyScoMotif [59] has been used to search for similar 3D structural motifs of these two motifs in newly characterized PdiTPSs and unreviewed terpene synthases, and the results indicate that these motifs are even present in PdiTPSs that produce new terpene scaffolds [82]. Furthermore, these two motifs can rectify incorrectly annotated terpene synthases obtained from UniProt. Supplementary Tables S7 and S8 contain information about these two motifs and the search results.

From the comprehensive results mentioned above, we can infer that these two structural motifs recur across PdiTPSs. This indicates the presence of strictly conserved feature modules around the substrates of PdiTPSs. Meanwhile, the relaxed residues around the substrates suggest their influence on catalytic specificity. It should be noted that the structures we employed are mostly predictions generated by AlphaFold2, which may not provide conformations with complete catalytic activity or accurately position side chains.

## 4. Conclusions

We have compiled and meticulously curated an extensive dataset of PdiTPSs with substrate–product pairs, sequences, and structural details, making it the most comprehensive resource of these enzymes to date. The dataset can facilitate in-depth sequence and structure analysis of PdiTPSs. There are some excellent examples, such as the virtual screening of P450 using characterized enzymes [83], product specificity analysis of sesquiterpene synthases [66], prediction of substrate classes for acyltransferases [84] and identification and classification of terpene synthase [85,86]. Furthermore, it serves as an essential repository of enzyme bioparts for combinatorial biology experiments to produce non-natural diterpene products and a broader spectrum of diterpene derivatives [62,87]. We attempt to categorize diterpene products by their skeletal structure. While this simplifies analysis and function comparison, it will encounter challenges when one enzyme produces multiple diterpene skeletons.

A strong correlation is observed between N-terminal subsequences and overall sequences, as well as the significant sequence differences between N- and C-terminal subsequences. Structural conservation demonstrates an increasing trend with a greater similarity between the N-terminal sequence and the overall sequence. Additionally, an independent topological structure exists between the local structure around the substrate and the overall structure. Quantitative analysis indicates that both sequence and structural similarities influence product distribution, though establishing a robust correlation remains elusive, underscoring the intricate relationships between sequence and structure in the functionality of PdiTPSs. Ultimately, our attention shifted to the examination of local structural features. This analysis allowed us to identify the spatial geometric arrangement of residues around substrates. Additionally, we assigned distinct potential functions to the conserved and less conserved residues. These conserved residues, in addition to the aspartic acid (D) in the DDXXD/DXDD, include tryptophan (W), phenylalanine (F), tyrosine (Y), methionine (M), arginine (R), serine (S), threonine (T), lysine (K), proline (P). These residues, along with aspartic acid (D), exhibit a stable distribution around the substrate of PdiTPSs. Furthermore, the spatial arrangement of these residues gives rise to distinctive 3D motifs in PdiTPSs I and PdiTPSs II, respectively. Contrastingly, if residues around a substrate exhibit high variability at the same spatial location, they tend to be product-specific residues. This correlation was supported by mutagenesis experiments involving previously reported product-specific residues.

It is noteworthy that AlphaFold2, renowned for its excellent stereochemical features [88], enables us to conduct hypothetical analyses of the overall and local structures of PdiTPSs. However, in some cases, limitations arise even when very high-confidence predictions differ from experimental maps on a global scale through distortion and domain orientation, and on a local scale in backbone and side-chain conformation [55,88]. Therefore, despite it has facilitated developments in the fields of biology and medicine [89], it cannot replace the results obtained from experiments and crystal structures. Considering its limitations, our structural analysis does not address intricate substrate-residue interactions and other factors like conformational changes in catalytic states [90,91]. In summary, AlphaFold2 can serve as a valuable tool to assist in hypothesis generation for experimental design and complement the interpretation of final experimental results. However, recognizing its limitations is crucial. Additionally, despite efforts to collect characterized PdiTPSs, insufficient data hinder normalization and omissions may exist. Further characterization of PdiTPSs remains essential for continuous optimization of computational studies. There is one more thing, 3D motifs, contingent on the molecular docking results, which may exhibit minor variations due to method and model choices. Despite rigorous manual checks, the complete elimination of such differences remains unattainable.

In conclusion, a comprehensive sequence-to-structure analysis of PdiTPSs adds to the current knowledge of diTPSs. The results highlight the spatial distribution of residues around the substrate of PdiTPSs, showcasing both conservation (3D motifs) and variation (less conserved residues) that contribute to the maintenance and diversification of PdiTPSs functionality. The identified local structure signatures around substrates prove beneficial for annotating diTPSs, offering valuable insights into a more comprehensive understanding of these enzymes from a structural perspective. Actually, the concept of 3D motifs, such as the well-known catalytic triad (Ser-His-Glu), has been proposed for a considerable time. Its widespread application, however, has been hindered by the challenges in obtaining a large number of reliable enzyme structures. The advent of AlphaFold2 and its continuous optimization have significantly improved this situation, allowing for effective analysis of enzyme product specificity through structural analysis. This approach has been extended to the large-scale annotation of enzyme functions [92,93], showcasing the advantages and potential of structural analysis in understanding PdiTPSs. Of course, our analysis has some disadvantages. Firstly, the 3D motifs obtained for PdiTPSs were not systematically compared with other terpene synthases. Future research could focus on a systematic comparison of 3D motifs in various classes of terpene synthases. Additionally, a comparative analysis of the similarities and differences in 3D motifs among terpene synthases producing diverse products could be explored. Secondly, the present method for obtaining 3D motifs was cumbersome and unsuitable for large datasets. The use of a faster approach, such as US-align, enables quick batch structure alignment and sequence output [94]. Visualizing these results with tools like Jalview [95] allows for swift identification of conserved and highly variable residue positions. These methods will enhance the efficiency of enzyme structure analysis.

## Figures and Tables

**Table 1:** PCC for PdiTPSs sequences, structures, and products.

Content	Pearson’s Correlation Coefficient
Local structures of 4 Å: Products	0.38
Local structures of 6 Å: Products	0.4
Local structures of 8 Å: Products	0.39
Local structures of 10 Å: Products	0.4
Overall structures: Products	0.36
C-subsequences: Products	0.35
N-subsequences: Products	0.54
NC-subsequences: Products	0.54
Overall structures: Products	0.55

Note: All *p* values < 0.001 in the table’s statistical results.

## Data Availability

Data is contained within the supplementary materials.

## Supplementary Materials

The following supporting information can be downloaded at: https://www.mdpi.com/article/10.3390/biom14010120/s1. Supplementary File S1, Supplementary File S2 [96,97,98,99,100,101,102,103,104,105,106,107,108,109,110,111,112,113,114,115,116,117,118,119,120,121,122,123,124,125,126,127,128,129,130,131,132,133,134,135,136,137,138,139,140,141,142,143,144,145,146,147,148,149,150,151,152,153,154].
