# Supplementary material for: Sequence-Structure Analysis Unlocking the Potential Functional Application of the Local 3D Motifs of Plant-Derived Diterpene Synthases
Source: Biomolecules. 2024 Jan 17;14(1):120. doi: 10.3390/biom14010120 (PMC10813164; doi:10.3390/biom14010120)
Supplement: Supplementary file 1 [file biomolecules-14-00120-s001.zip › Supplementary File S1.pdf]

Supporting Information

Sequence-Structure Analysis Unlocking the Potential Functional Application of the Local 3D Motifs of Plant-Derived Diterpene Synthases

Yalan Zhao [1,2,†], Yupeng Liang [1,2,†], Gan Luo [1,2], Yi Li [3,*], Xiulin Han [1,2] and Mengliang Wen [1,2,*]

1 *National Key Laboratory for Conservation and Utilization of Bio-Resources in Yunnan, Yunnan University, Kunming 650091, China*

2 *Key Laboratory of Microbial Diversity in Southwest China, Ministry of Education, Yunnan Institute of Microbiology, School of Life Sciences, Yunnan University, Kunming 650091, China*

3 *College of Mathematics and Computer Science, Dali University, Dali 671003, China*

* *Correspondence: yili@dali.edu.cn (Y.L.); mlwen@ynu.edu.cn (M.W.)*

† *These authors contributed equally to this work.*

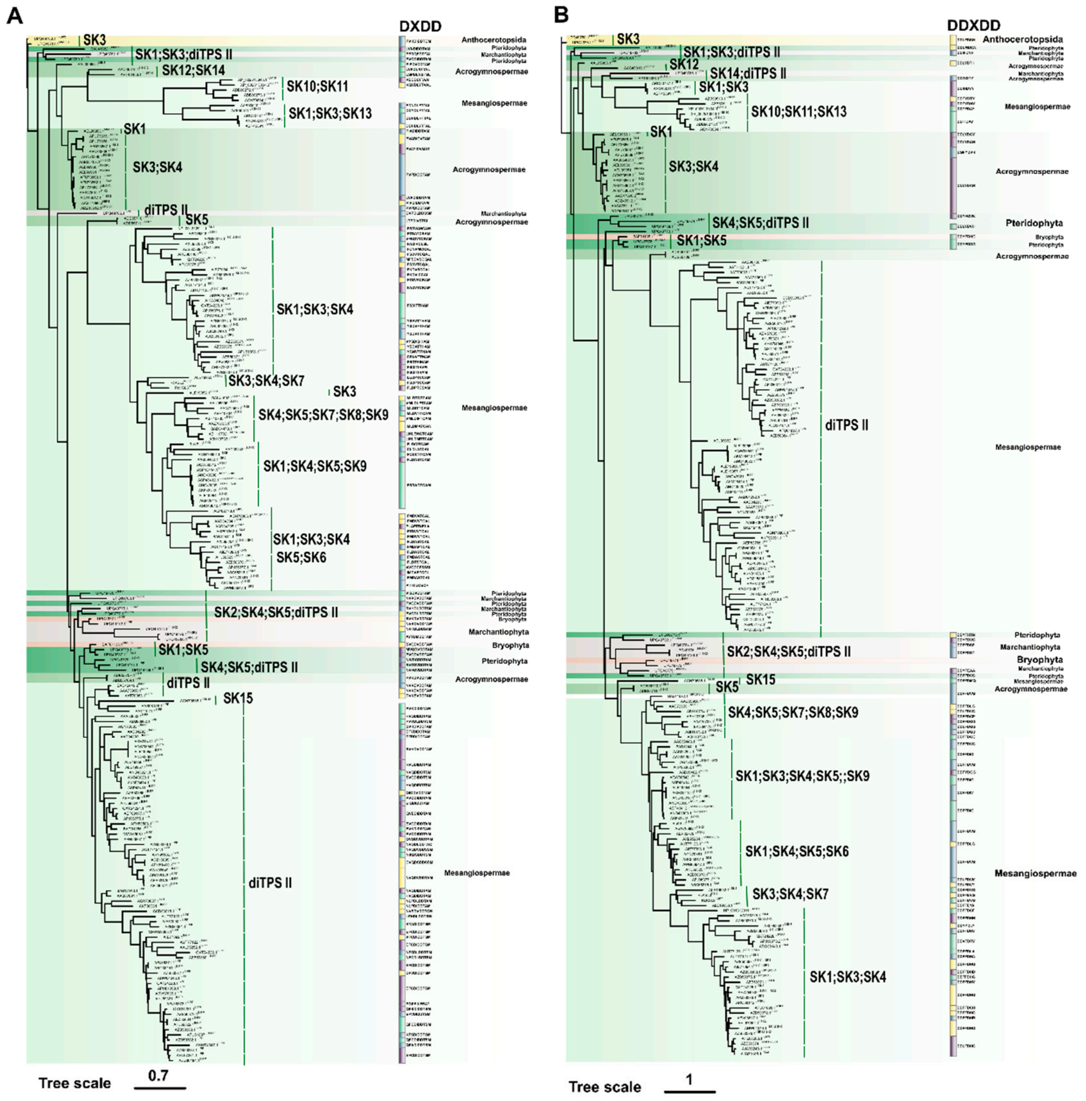

Figure S1. The relationship between the phylogeny of the N-terminal and C-terminal domains of PdiTPSs the motifs related to product skeleton and function, and the classification level of enzyme sources. The phylogenetic tree indicates the ID, product type, and product skeleton classification of each PdiTPSs. **A**) N-terminal domain, DXDD is the signature motif of the N-terminal domain; **B**) C-terminal domain, DDXXD motif is the signature motif of the C-terminal domain. The extended motifs of these two motifs are shown here.

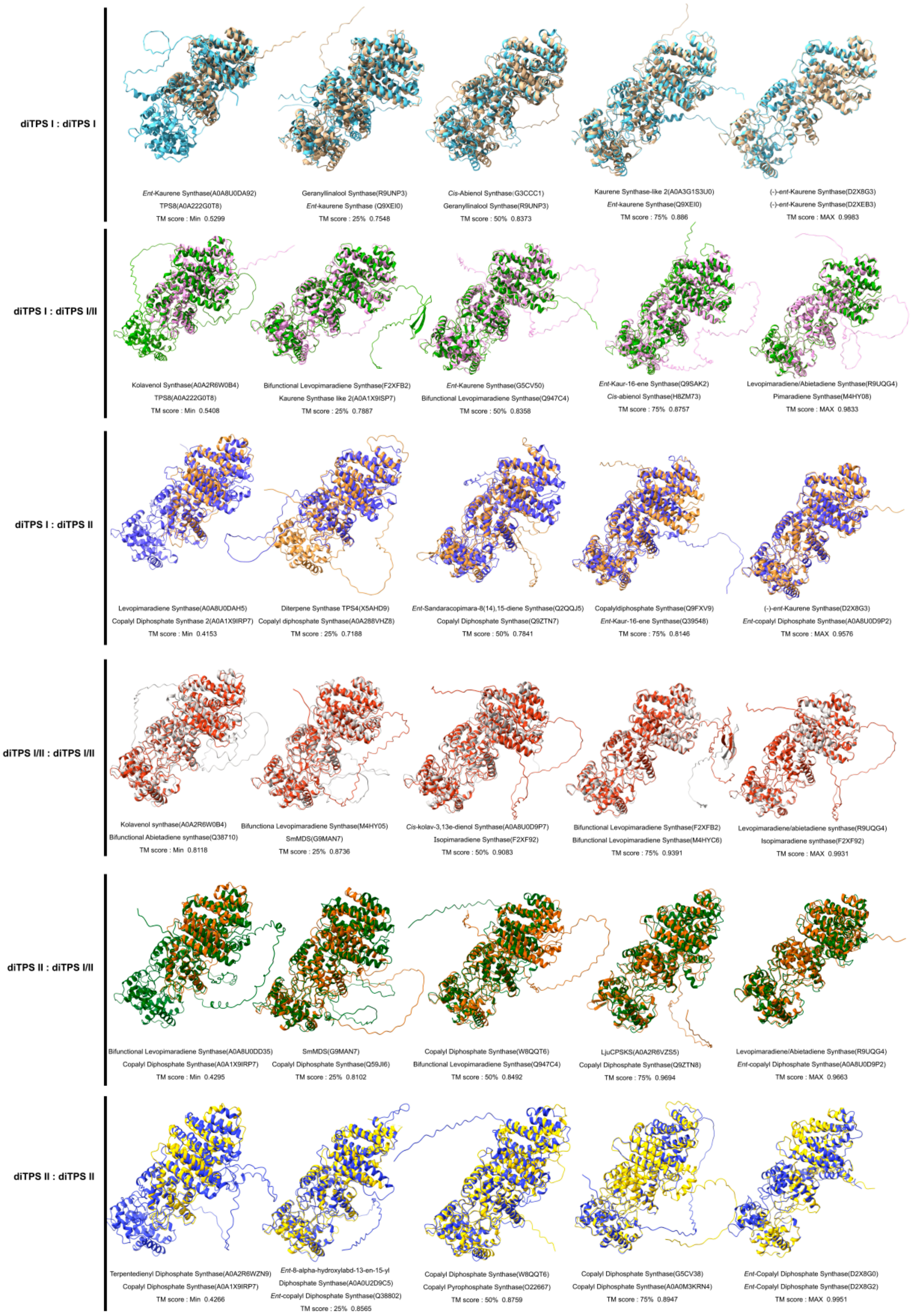

Figure S2. Representative structural superpositions of the minimum, 25% (Q1), 50% (Q2), 75% (Q3), and maximum observed values of the distribution of topological similarity between PdiTPSs I, PdiTPSs II, and PdiTPSs I/II.

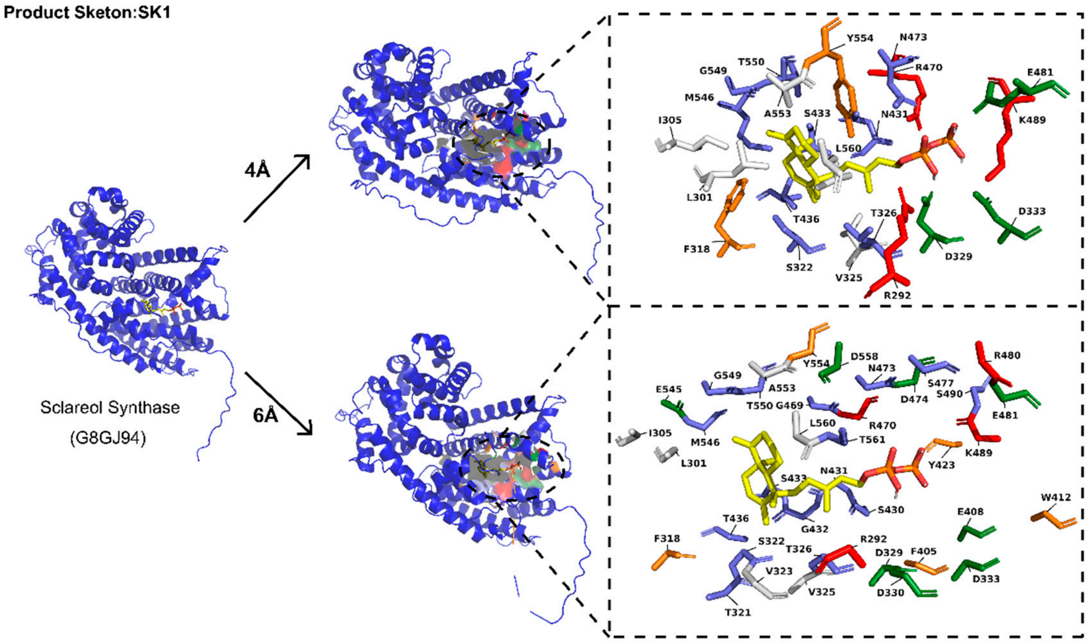

Figure S3. Specific residue structures and topology formed by residues at 4Å and 6Å radial distances from the substrate of PdiTPSs producing the SK1 skeleton type.

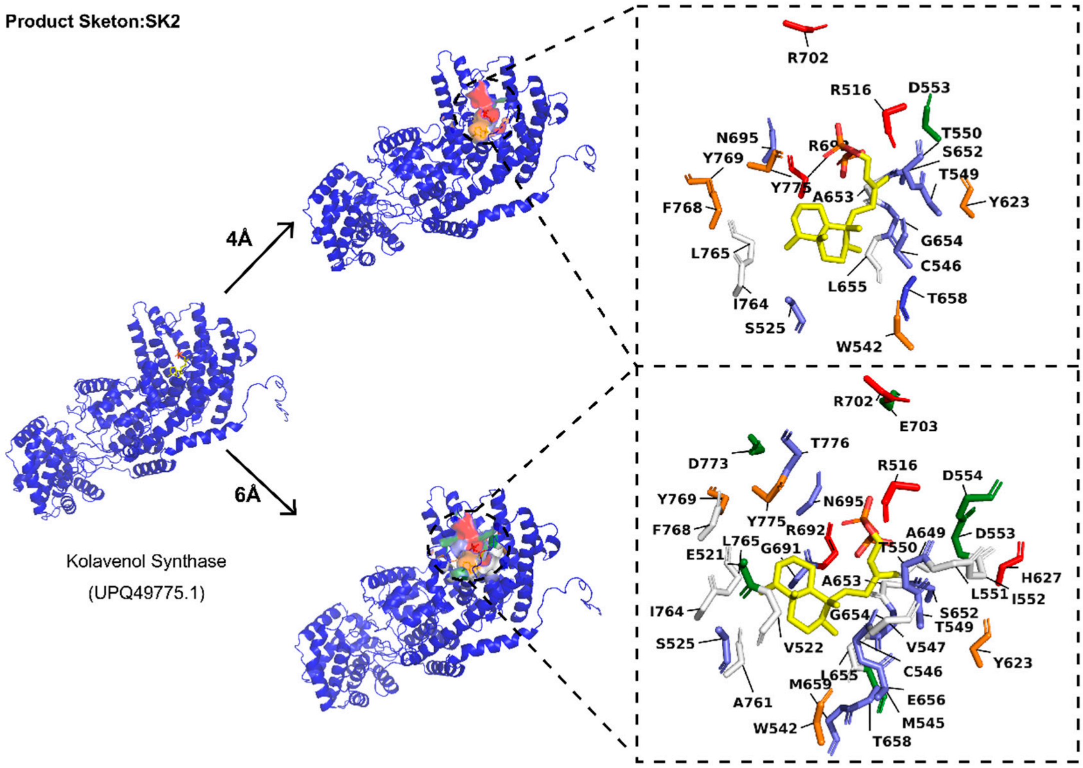

Figure S4. Specific residue structures and topology formed by residues at 4Å and 6Å radial distances from the substrate of PdiTPSs producing the SK2 skeleton type.

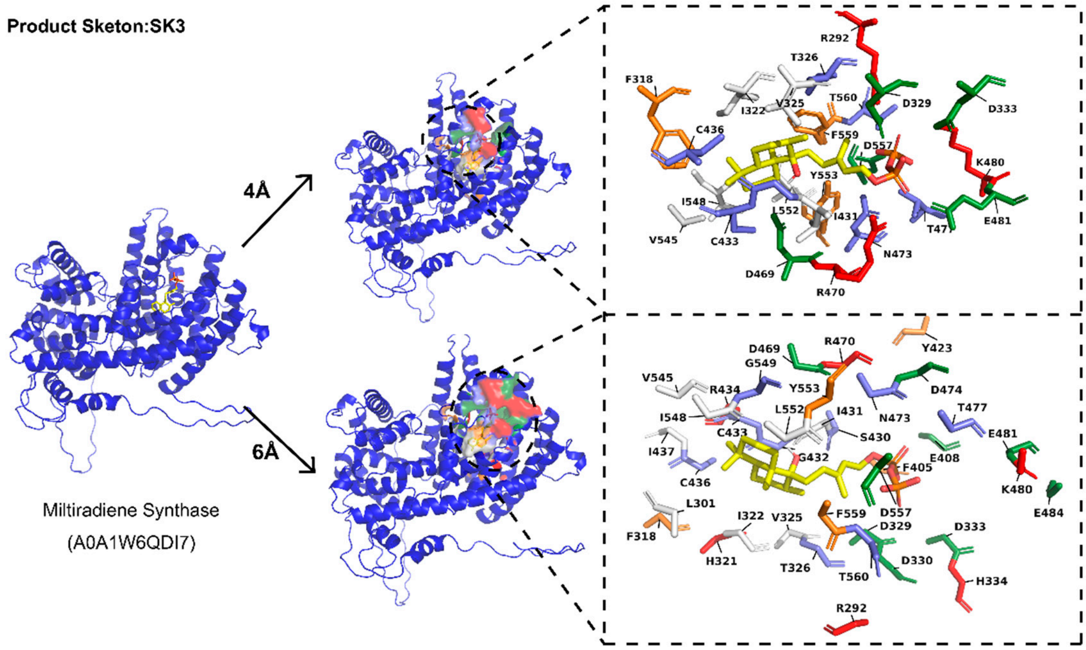

Figure S5. Specific residue structures and topology formed by residues at 4Å and 6Å radial distances from the substrate of PdiTPSs producing the SK3 skeleton type.

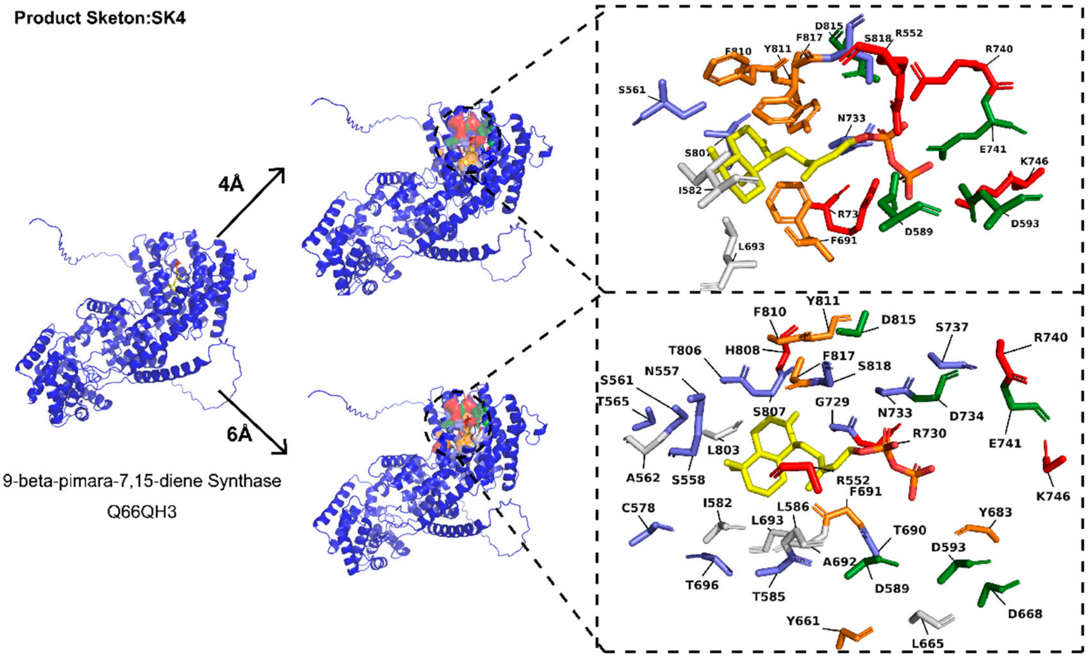

Figure S6. Specific residue structures and topology formed by residues at 4Å and 6Å radial distances from the substrate of PdiTPSs producing the SK4 skeleton type.

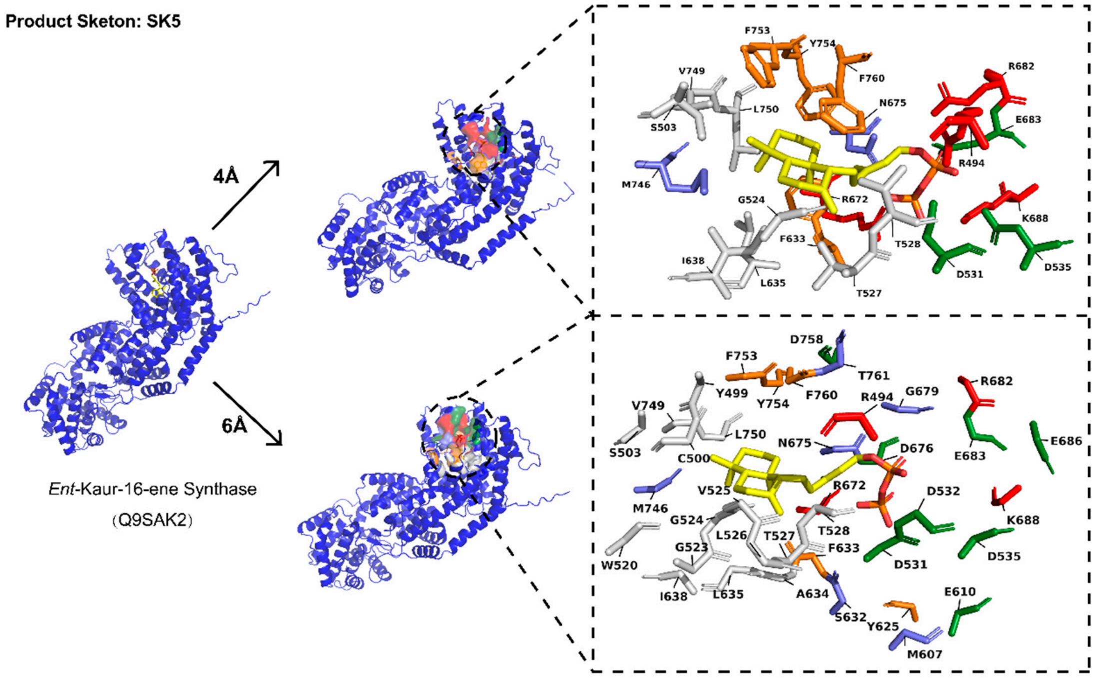

Figure S7. Specific residue structures and topology formed by residues at 4Å and 6Å radial distances from the substrate of PdiTPSs producing the SK5 skeleton type.

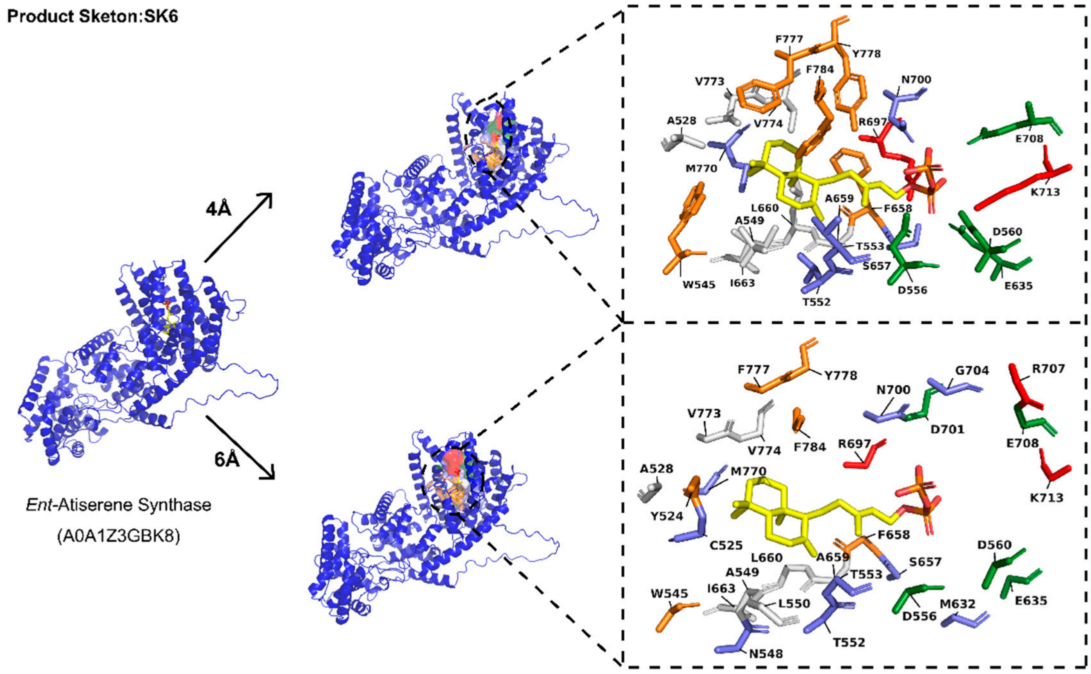

Figure S8. Specific residue structures and topology formed by residues at 4Å and 6Å radial distances from the substrate of PdiTPSs producing the SK6 skeleton type.

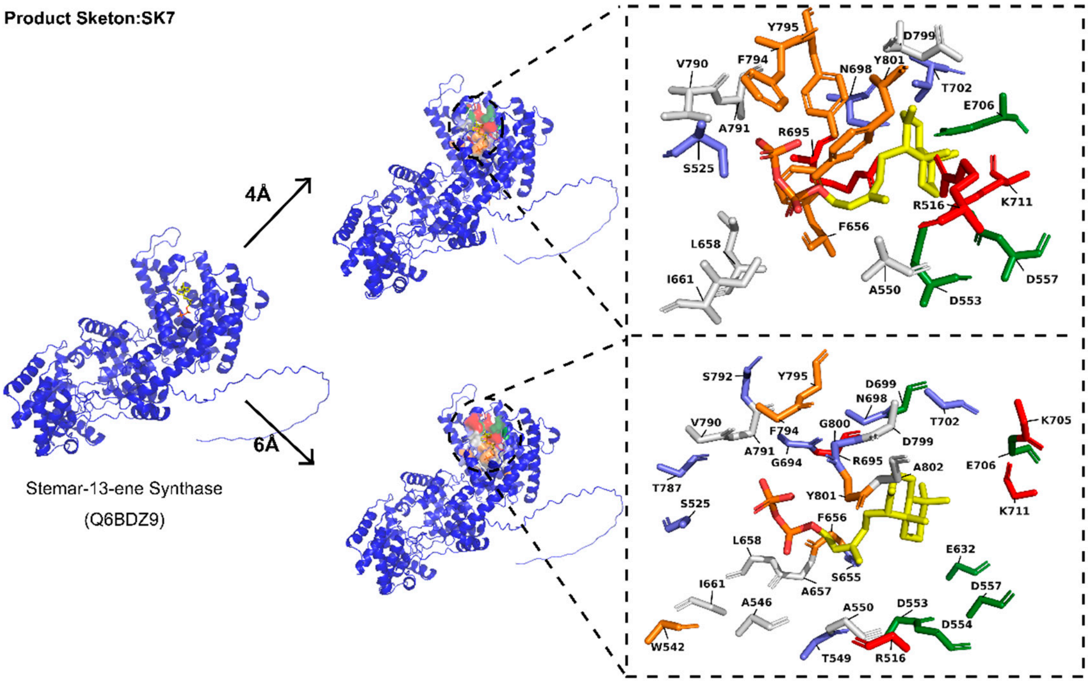

Figure S9. Specific residue structures and topology formed by residues at 4Å and 6Å radial distances from the substrate of PdiTPSs producing the SK7 skeleton type.

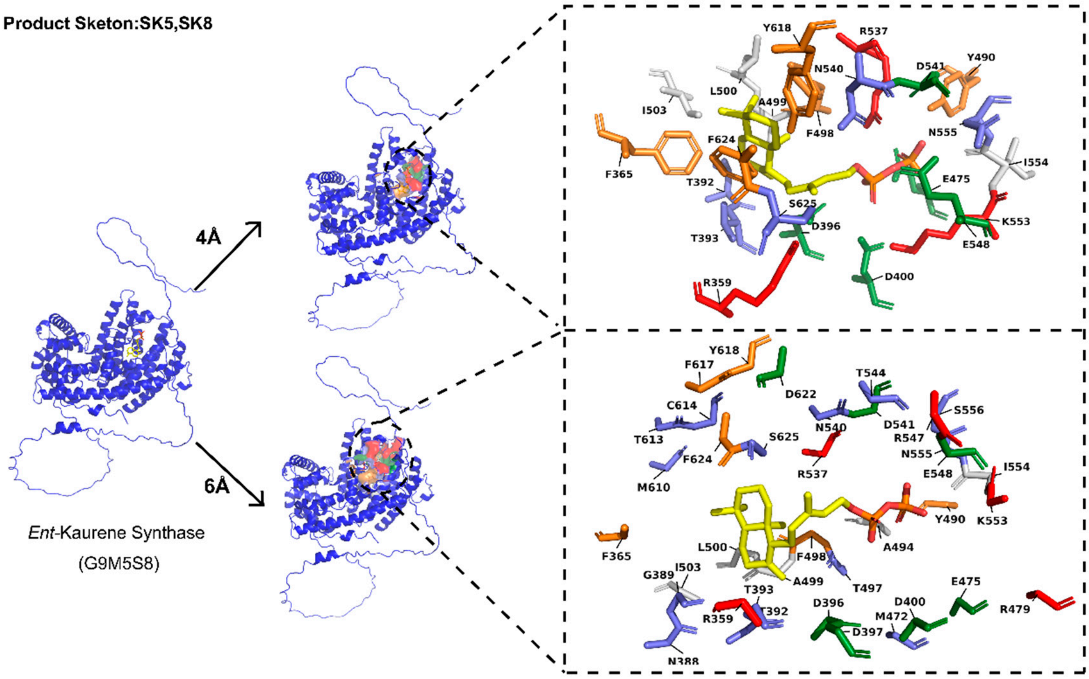

Figure S10. Specific residue structures and topology formed by residues at 4Å and 6Å radial distances from the substrate of PdiTPSs producing the SK8 skeleton type.

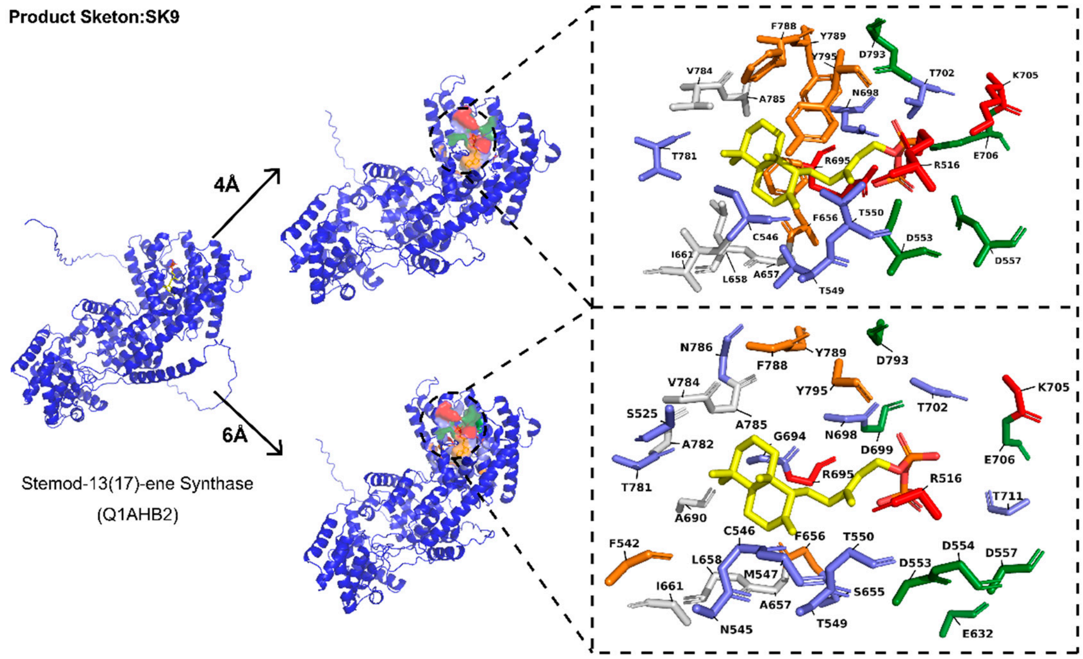

Figure S11. Specific residue structures and topology formed by residues at 4Å and 6Å radial distances from the substrate of PdiTPSs producing the SK9 skeleton type.

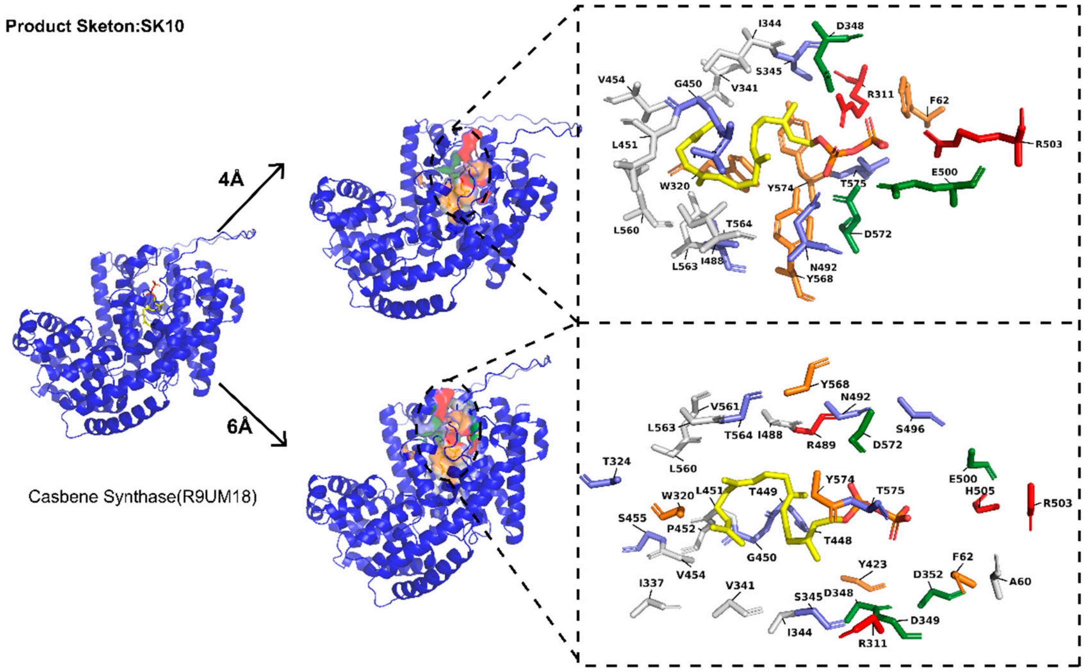

Figure S12. Specific residue structures and topology formed by residues at 4Å and 6Å radial distances from the substrate of PdiTPSs producing the SK10 skeleton type.

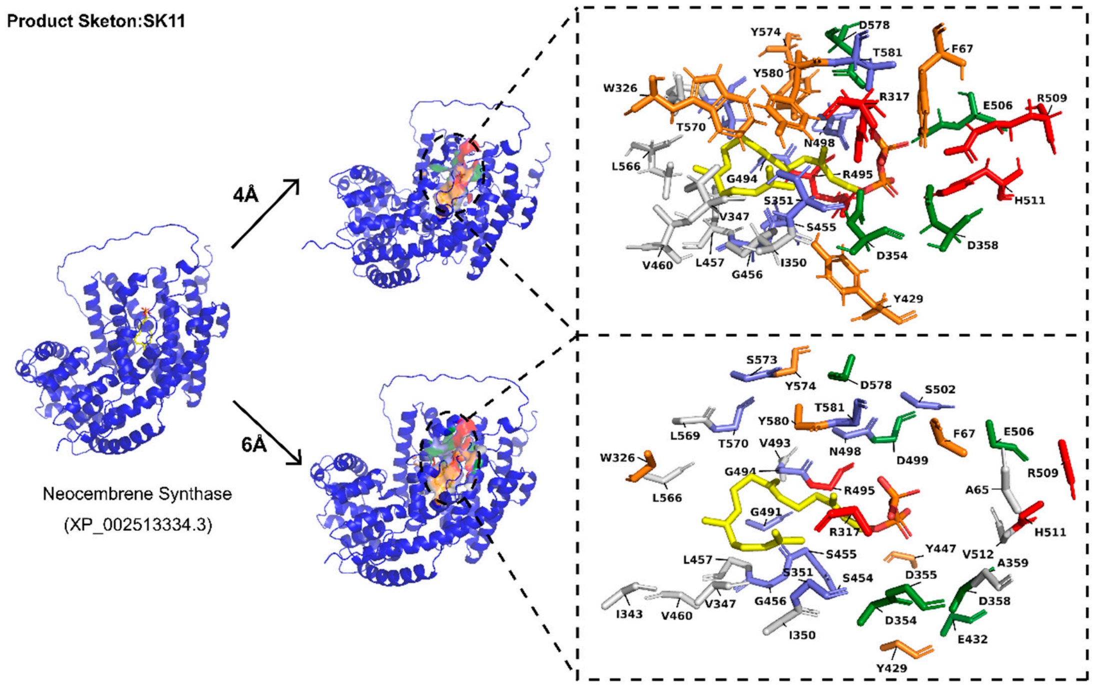

Figure S13. Specific residue structures and topology formed by residues at 4Å and 6Å radial distances from the substrate of PdiTPSs producing the SK11 skeleton type.

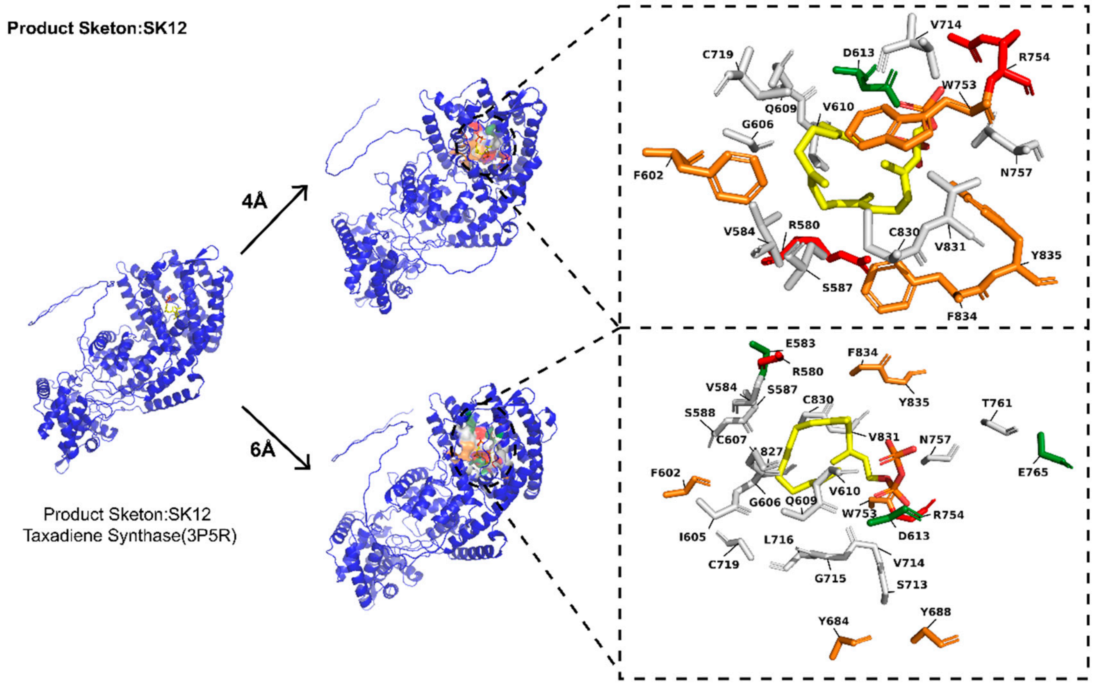

Figure S14. Specific residue structures and topology formed by residues at 4Å and 6Å radial distances from the substrate of PdiTPSs producing the SK12 skeleton type.

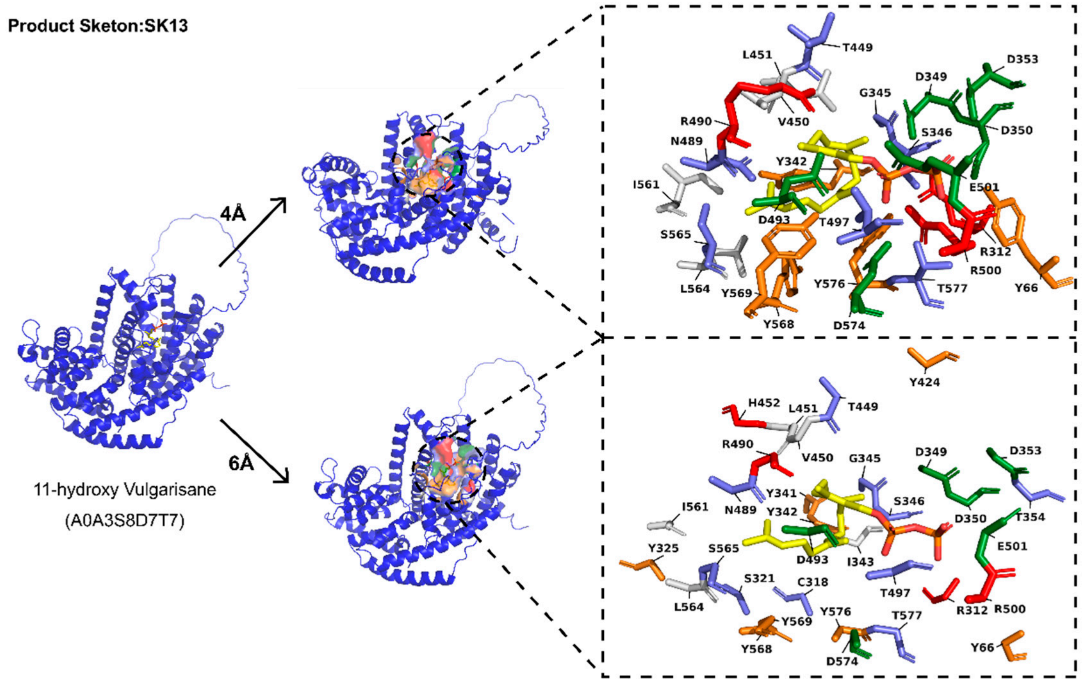

Figure S15. Specific residue structures and topology formed by residues at 4Å and 6Å radial distances from the substrate of PdiTPSs producing the SK13 skeleton type.

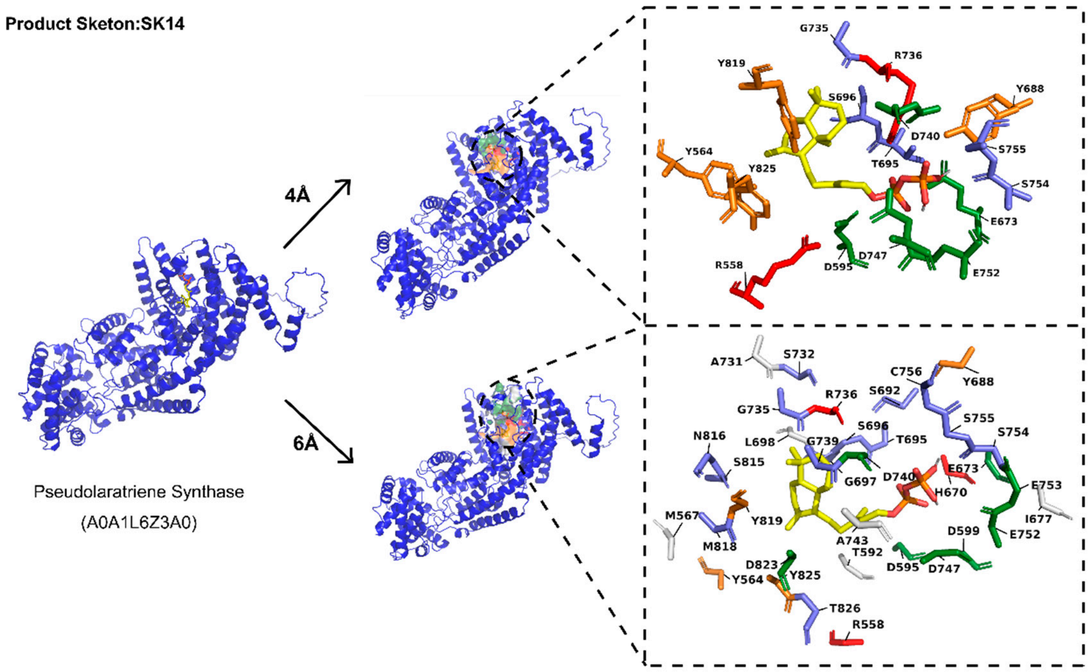

Figure S16. Specific residue structures and topology formed by residues at 4Å and 6Å radial distances from the substrate of PdiTPSs producing the SK14 skeleton type.

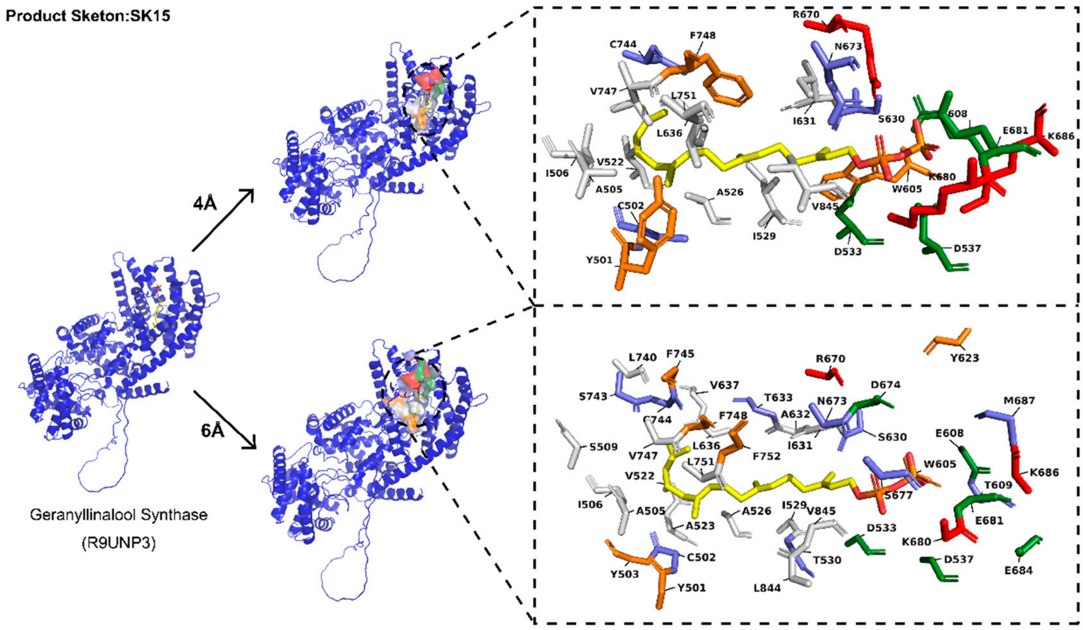

Figure S17. Specific residue structures and topology formed by residues at 4Å and 6Å radial distances from the substrate of PdiTPSs producing the SK15 skeleton type.

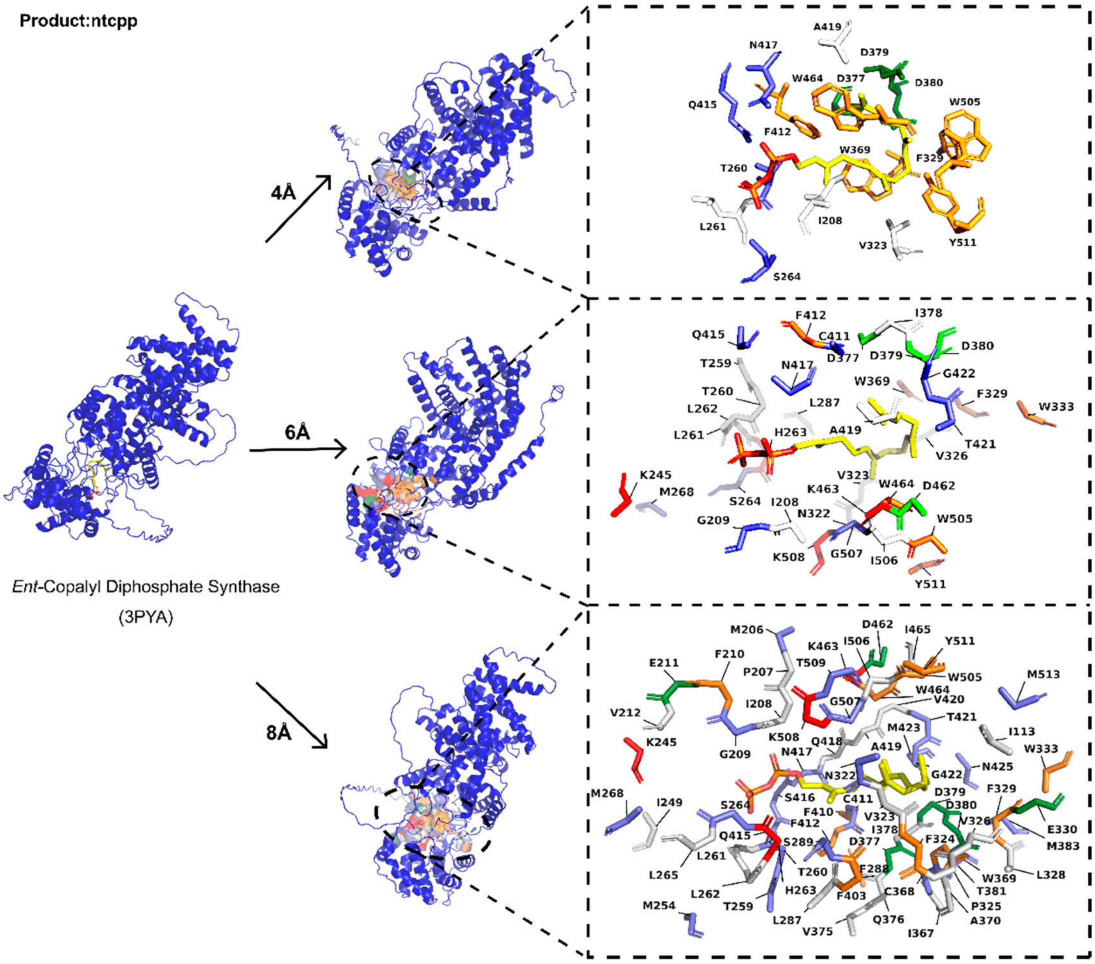

Figure S18. Topological structure and specific residues within 4Å, 6Å, and 8Å radial of the substrate of PdiTPSs, which produce ntcpp and have confirmed functional residues.

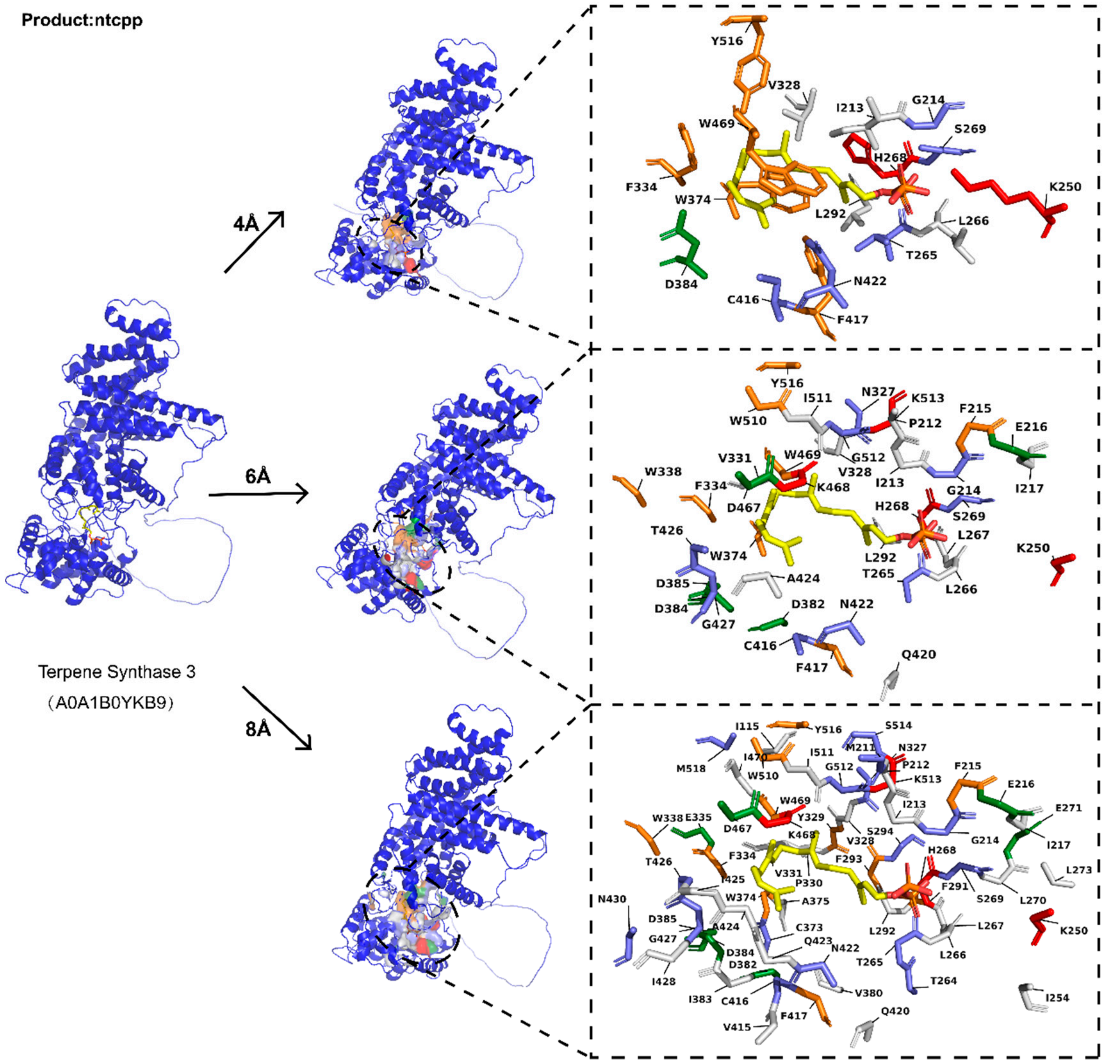

Figure S19. Topological structure and specific residues within 4Å, 6Å, and 8Å radial of the substrate of PdiTPSs, which produce ntcpp and have confirmed functional residues.

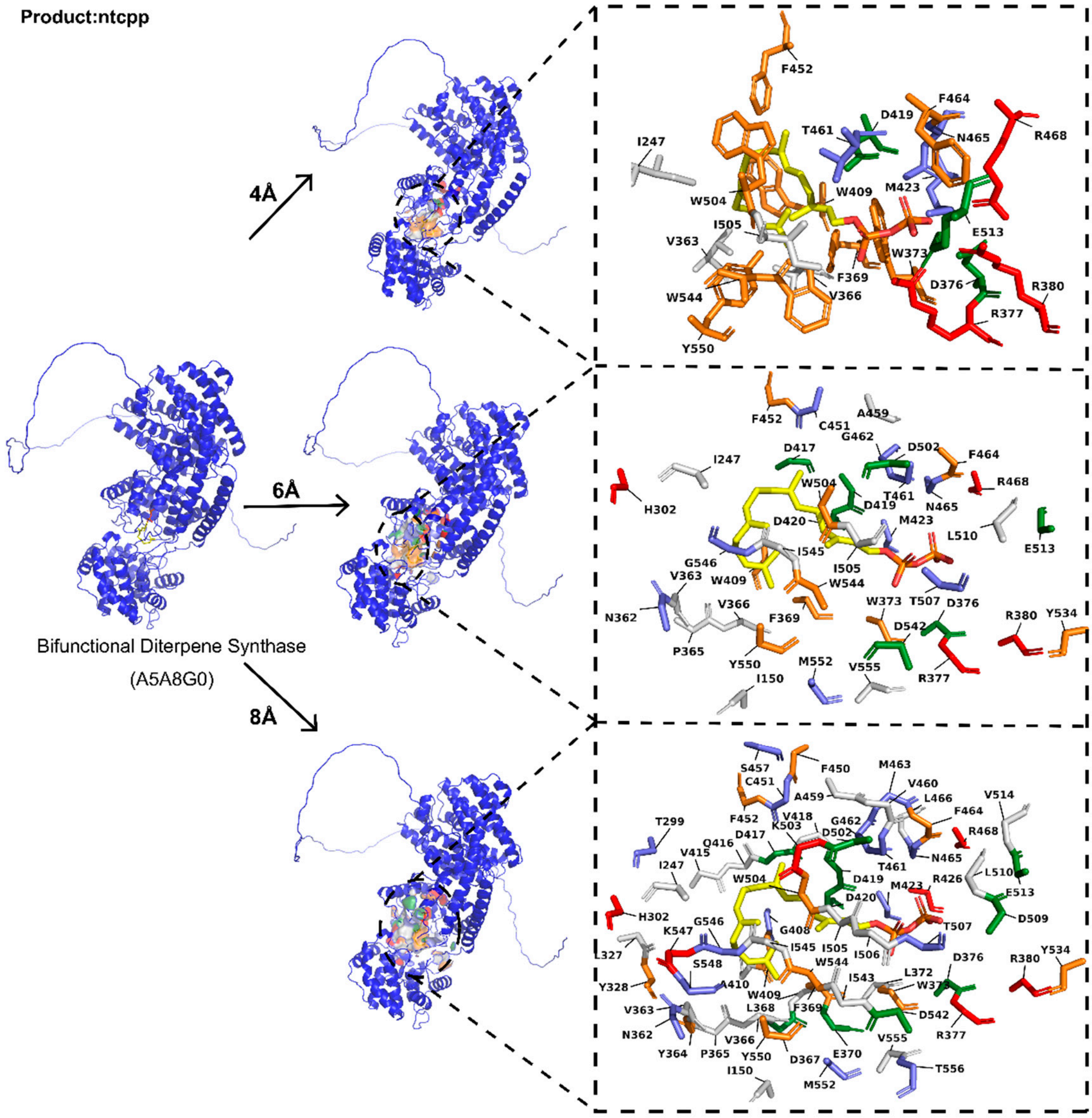

Figure S20. Topological structure and specific residues within 4Å, 6Å, and 8Å radial of the substrate of PdiTPSs, which produce ntcpp and have confirmed functional residues.

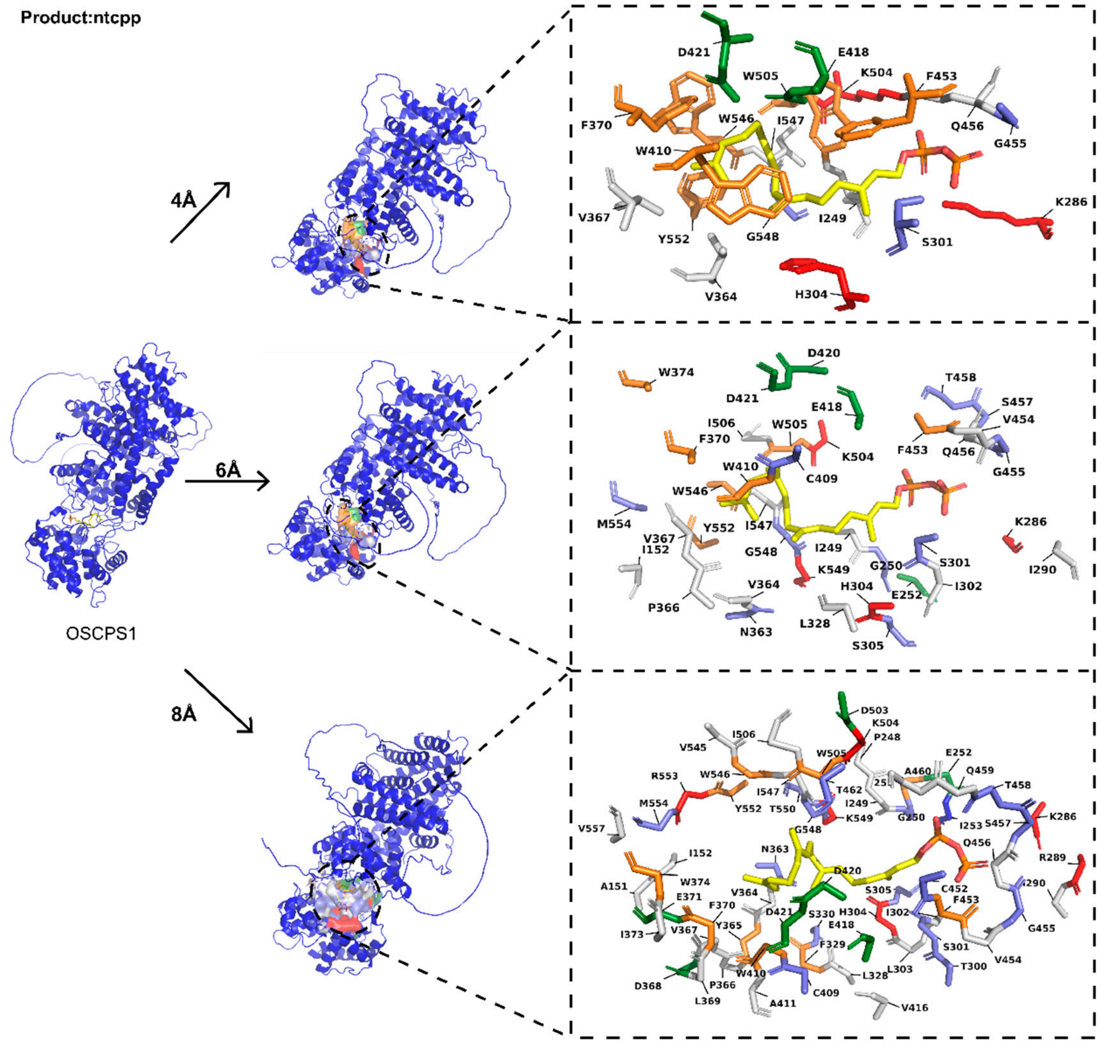

Figure S21. Topological structure and specific residues within 4Å, 6Å, and 8Å radial of the substrate of PdiTPSs, which produce ntcpp and have confirmed functional residues.

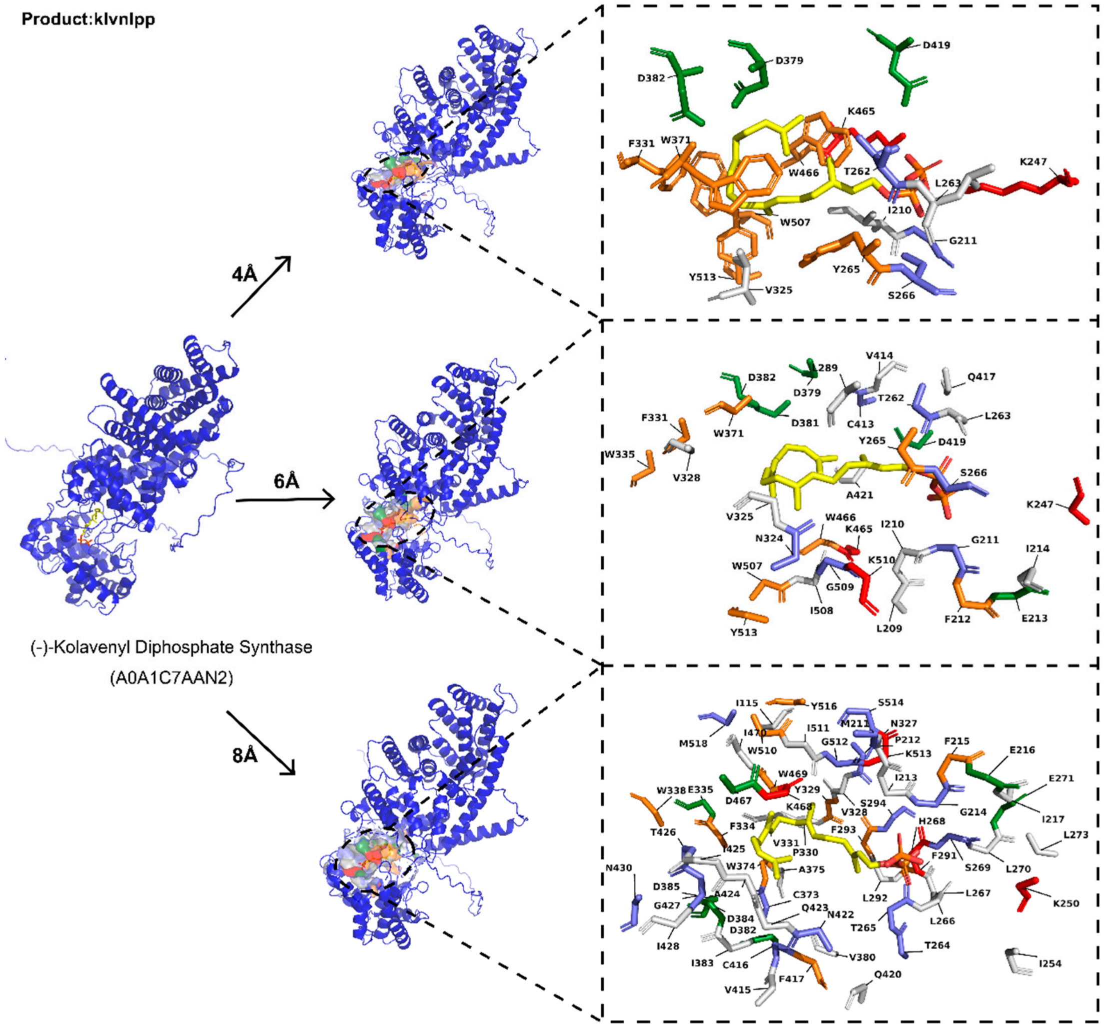

Figure S22. Topological structure and specific residues within 4Å, 6Å, and 8Å radial of the substrate of PdiTPSs, which produce klvnlpp and have confirmed functional residues.

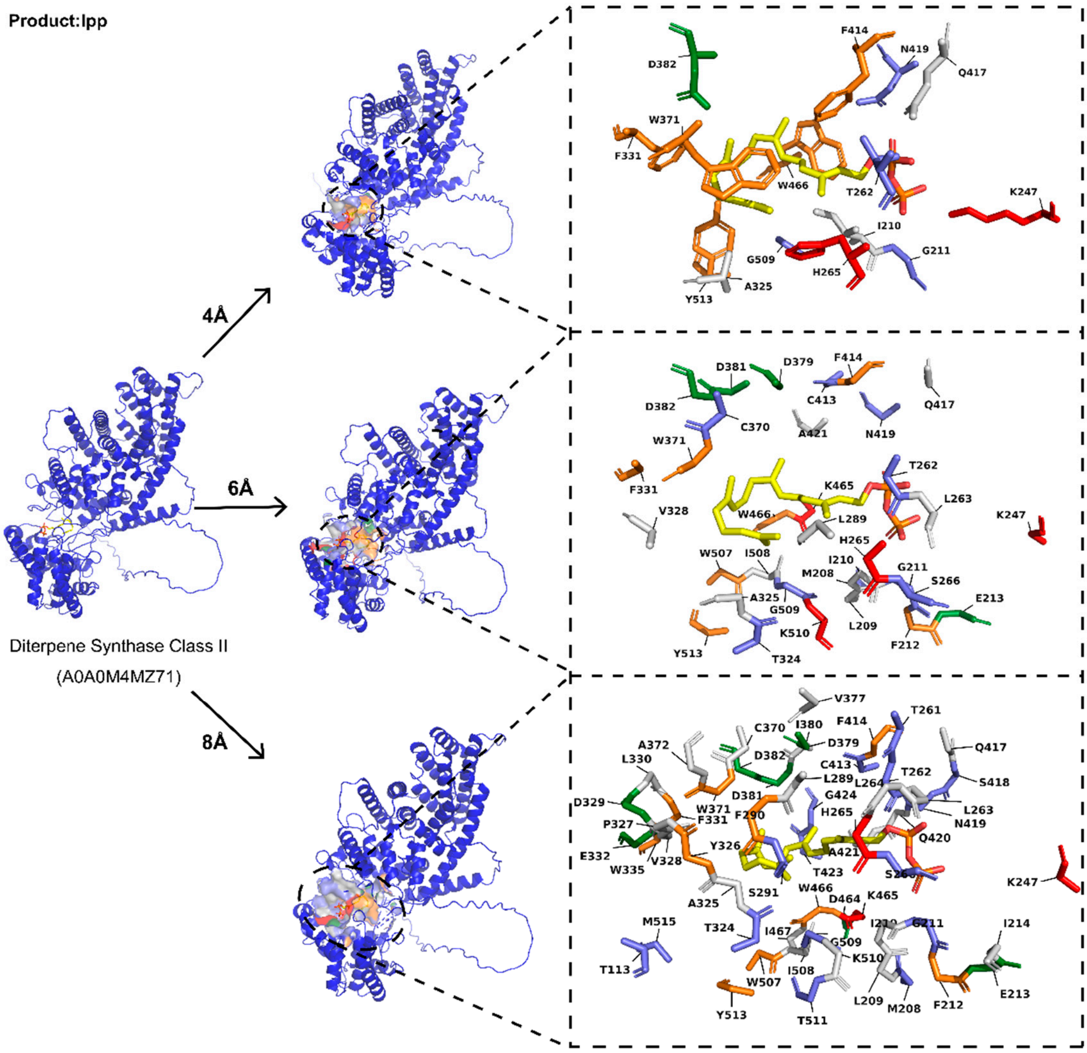

Figure S23. Topological structure and specific residues within 4Å, 6Å, and 8Å radial of the substrate of PdiTPSs, which produce lpp and have confirmed functional residues.

Table S9 Pearson's correlation coefficients between different sequence types and structures of PdiTPSs

Content	*Pearson's correlation coefficients*
Nsequence : 4 Å radial	0.4
Nsequence : 6 Å radial	0.36
Nsequence : 8 Å radial	0.34
Nsequence : 10 Å radial	0.33
Nsequence : Overall structure	0.63
Csequence : 4 Å radial	0.55
Csequence : 6 Å radial	0.55
Csequence : 8 Å radial	0.52
Csequence : 10 Å radial	0.54
Csequence : Overall structure	0.34
NCsequence : 4 Å radial	0.6
NCsequence : 6 Å radial	0.59
NCsequence : 8 Å radial	0.55
NCsequence : 10 Å radial	0.56
NCsequence : Overall structure	0.55
Overall sequence : 4 Å radial	0.51
Overall sequence : 6 Å radial	0.46
Overall sequence : 8 Å radial	0.42
Overall sequence : 10 Å radial	0.42
Overall sequence : Overall structure	0.7

Note: All p values in the table's statistical results are < 0.001.
